# Clarifying the composition of the ATP consumption factors required for maintaining ion homeostasis in mouse rod photoreceptors

**DOI:** 10.1038/s41598-023-40663-y

**Published:** 2023-08-29

**Authors:** Yuttamol Muangkram, Yukiko Himeno, Akira Amano

**Affiliations:** Department of Bioinformatics, College of Life Sciences, Ritsumeikan University, Shiga, Japan

**Keywords:** Biophysics, Cell biology, Computational biology and bioinformatics, Molecular biology, Neuroscience, Physiology

## Abstract

To date, no effective treatment has been established for photoreceptor loss due to energy imbalances, but numerous therapeutic approaches have reported some success in slowing photoreceptor degeneration by downregulating energy demand. However, the detailed mechanisms remain unclear. This study aimed to clarify the composition of ATP consumption factors in photoreceptors in darkness and in light. We introduced mathematical formulas for ionic current activities combined with a phototransduction model to form a new mathematical model for estimating the energy expenditure of each ionic current. The proposed model included various ionic currents identified in mouse rods using a gene expression database incorporating an available electrophysiological recording of each specific gene. ATP was mainly consumed by Na^+^/K^+^-ATPase and plasma membrane Ca^2+^-ATPase pumps to remove excess Na^+^ and Ca^2+^. The rod consumed 7 $\times$ 10^7^ molecules of ATP s^−1^, where 65% was used to remove ions from the cyclic nucleotide-gated channel and 20% from the hyperpolarization-activated current in darkness. Increased light intensity raised the energy requirements of the complex phototransduction cascade mechanisms. Nevertheless, the overall energy consumption was less than that in darkness due to the significant reduction in ATPase activities, where the hyperpolarization-activated current proportion increased to 83%. A better understanding of energy demand/supply may provide an effective tool for investigating retinal pathophysiological changes and analyzing novel therapeutic treatments related to the energy consumption of photoreceptors.

## Introduction

It is well known that the retina is one of the most energy-consuming tissues with the highest oxygen demand in the human body. Most of the energy is mainly spent on photoreceptor metabolic requirements. However, the detailed mechanisms are less well understood. It is generally agreed that photoreceptor energy demand in darkness is greater than that in light conditions. ATP expenditure by rods is reported to be approximately 9 $\times$ 10^7^ molecules of ATP s^−1^ in darkness and 2 $\times$ 10^7^ molecules of ATP s^−1^ in bright illumination^1^. Energy is predominantly consumed via the Na^+^/K^+^ ATPase pump (*I*_NaK_) and plasma membrane Ca^2+^ ATPase pump (*I*_PMCA_) for removing excess Na^+^ and Ca^2+^, respectively. One ATP molecule is hydrolyzed to transport three Na^+^ ions out and two K^+^ ions into the cell through *I*_NaK_, whereas *I*_PMCA_ has a stoichiometry of 1:1, i.e., one Ca^2+^ per hydrolyzed ATP.

The initial steps of vision represented by photoreceptors via light-induced isomerization of 11-*cis*-retinal to all-*trans*-retinal trigger the phototransduction cascade and the subsequent decline in cGMP concentration, leading to the closure of light-sensitive current and neuronal hyperpolarization. It has been suggested that the energy expenditure of the complex transduction cascades at saturating illumination is relatively small compared to the total energy consumption in darkness^1^. This evidence may support the view that photoreceptors use most of their energy to maintain ion homeostasis via *I*_NaK_ and *I*_PMCA_ in darkness and in light. Understanding the fundamentals of ion homeostatic regulatory mechanisms may allow for the development of effective treatment strategies for retinal diseases related to photoreceptor energy consumption.

Photoreceptors absorb nutrients and oxygen to generate energy from the retinal pigment epithelium. Mitochondrial oxidative phosphorylation is the primary fuel source for photoreceptors. A continuous O_2_ supply is essential for photoreceptors because it cannot be stored. A disruption in the balance between oxygen supply and energy demand impairs oxidative phosphorylation, leading to many retinal diseases, such as retinitis pigmentosa, age-related macular degeneration, and retinal detachment. Rods are more sensitive than cones. Morphological abnormalities in human rods can be observed after age 30^2^. In age-related macular degeneration, rods are affected earlier and more seriously than cones^3^. A decrease in mitochondria reduces the cellular membrane potential and energy supply, leading to increased reactive oxygen species and photoreceptor loss^4^. To date, no effective treatment has been established for photoreceptor loss, but numerous therapeutic approaches have reported some success in slowing photoreceptor degeneration by downregulating energy demand.

In attempts to improve photoreceptor survivability and prevent vision loss, several studies have reported some neuroprotective therapies, such as an adenosine monophosphate-activated protein kinase activator for preserving ATP levels under photostress^5^, a ketogenic and low-protein diet for slowing retinal degeneration^6^, and nicotinamide mononucleotide for restoring retinal detachment^7^. Furthermore, some studies have shown improvement in mitochondrial function and reduction in cell loss by long wavelengths absorbed in mitochondrial respiration^4,8^. A better understanding of the crucial energy demand in photoreceptors, i.e., ionic current activities, may provide an effective tool for investigating retinal pathophysiological changes and analyzing novel therapeutic treatments related to the energy consumption of photoreceptors.

Mathematical models have provided extensive insights into complex biophysiological phenomena and are widely applied to describe clinical and experimental studies. Several conductance-based models of rod photoreceptors have well described the changes in electrical properties, ionic currents, and light-sensitive currents in response to light in lower vertebrate photoreceptors^9–11^. However, the alterations in intracellular ion concentrations essential for evaluating each ionic current’s driving force and estimating the energy expenditure via *I*_NaK_ and *I*_PMCA_ have not been detailed. Furthermore, electrophysiological and morphological differences exist between lower vertebrate and mammalian rod photoreceptors.

In this preliminary study, we aimed to clarify the composition of the ATP consumption factors of mouse rod photoreceptors in darkness and in light. The mathematical formulas for ionic current activities combined with a phototransduction model were introduced to form a new mathematical model for estimating the energy expenditure of each ionic current. The proposed model should provide a better understanding of the critical role of physiological phenomena with detailed mechanisms that could explain how photoreceptors maintain ion homeostasis in response to light and darkness.

## Methods

### Model characteristics

The genes encoding ion channels in mouse rods have been revealed using single-cell RNA-Seq analysis^12^. The proposed model consisted of various ion channels, ion pumps, exchangers, and transporters identified using a gene expression database incorporating an available electrophysiological recording of each specific gene (Table 1).

**Table 1:** Various ion channels, ion pumps, exchangers, and transporters analyzed in the current study.

Symbol	Ionic currents	Genes
*I*_CNG_	Cyclic nucleotide-gated ion channel	*Cnga1/Cngb1*^13^
*I*_NCKX_	Potassium-dependent sodium/calcium exchanger	*Slc24a1*
*I*_CaL_	L-type voltage-gated calcium channel	*Cacna1f*^14–17^
*I*_h_	Hyperpolarization-activated channel	*Hcn1*^11,17–19^
*I*_Kv_	Voltage-gated potassium channel	*Kcnb1/Kcnv2*^20–23^
*I*_KCa_	Calcium-dependent potassium channel	*Kcnma1,Kcnn1*^24,25^
*I*_ClCa_	Calcium-dependent chloride channel	*Ano2*^17,26,27^
*I*_NaK_	Sodium/potassium-ATPase pump	*Atp1a3/Atp1b2*
*I*_PMCA_	Plasma membrane Ca^2+^-ATPase pump	*Atp2b1*
*I*_NCX_	Sodium/calcium exchanger	*Slc8a3*
*I*_NKCC1_	Sodium/potassium/chloride cotransporter	*Slc12a2*
*I*_KCC2_	Chloride/potassium cotransporter	*Slc12a5*
*I*_L_	Unidentified leak currents	

**Figure 1:**
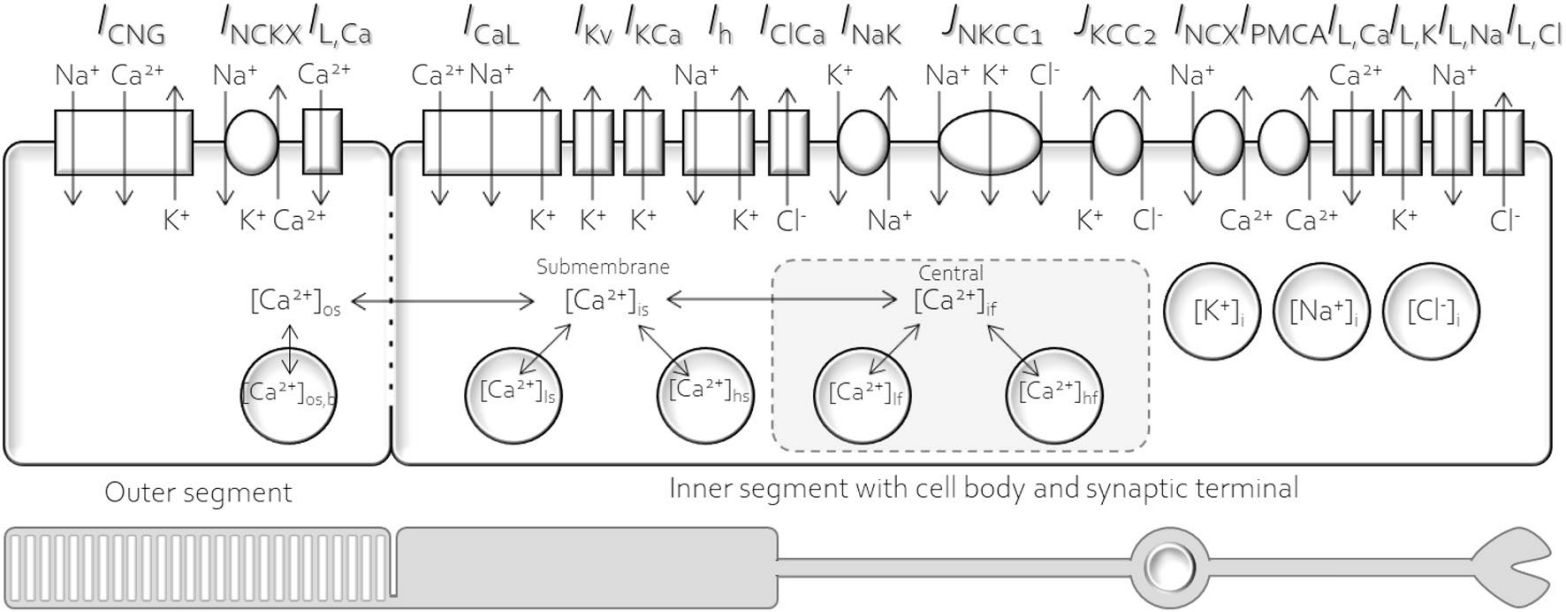
Schematic of the mouse rod photoreceptor model in the current study. (Upper) various ion channels, ion pumps, exchangers, transports, intracellular calcium system, and essential ion species (K^+^, Na^+^, and Cl^−^), (Lower) two main compartments: (1) outer segment and (2) inner segment with cell body and synaptic terminal.

Rod photoreceptors consist of four parts: outer segment, inner segment, cell body, and synaptic terminal. For simplification, as in earlier studies^9,10^, the rod photoreceptor in the current research is divided into two compartments: (1) outer segment and (2) inner segment with cell body and synaptic terminal (Fig. 1).

The cyclic nucleotide-gated ion channel (*I*_CNG_) and potassium-dependent sodium/calcium exchanger (*I*_NCKX_) are only found in the plasma membrane of the outer segment. *I*_CNG_, a nonselective cation channel with no significant anion permeability, is controlled by the light-sensitive conductance of two messenger molecules, cGMP and Ca^2+^. *I*_CNG_ forms heterotetrameric complexes composed of three *Cnga1* subunits and one *Cngb1* subunit^28^, mediating Na^+^ and Ca^2+^ influxes and K^+^ efflux. The permeability ratios of Na^+^ relative to K^+^ and Na^+^ relative to Ca^2+^ are approximately 1 and 2–6, respectively^29–32^. *I*_NCKX_ has a stoichiometry of exchange of four Na^+^ for one Ca^2+^ and one K^+^ and plays a vital role in maintaining Ca^2+^ efflux function in the outer segment. The homomeric channel of *I*_NCKX_ is formed by *Slc24a1* genes or NCKX1 proteins strongly expressed in mouse rods^33^. The amplitude of *I*_NCKX_ in salamander photoreceptors is ~ 10% of *I*_CNG_, and it has been suggested that it is similar to that of mice^17^.

Earlier studies have revealed at least five types of ion channels identified in the inner segment and synaptic terminal of amphibian photoreceptors, including hyperpolarization-activated channel (*I*_h_), delayed rectifying K^+^ channel (*I*_Kx_), voltage-gated Ca^2+^ channel (*I*_Ca_), Ca^2+^-dependent K^+^ channel (*I*_KCa_), and Ca^2+^-dependent Cl^-^ channel (*I*_ClCa_)^34–36^. We assumed that the electrophysiological behaviors of each animal species might have a similar pattern if the genes encoding ion channels have no fundamentally different gene expression profiles. *I*_h_ is encoded by the *Hcn1* gene, which is strongly expressed in mouse rods^12,23^. *Hcn1* forms a functional homomeric channel for Na^+^ influx and K^+^ efflux with a relative permeability ratio for Na^+^ and K^+^ (*P*_Na_/*P*_K_) of 0.3–0.36^37–39^. This channel has a vital role in shaping the initial transient in the membrane hyperpolarization of rod photoreceptors in response to light. *I*_Kx_ or noninactivating K^+^ channels have recently been classified as voltage-gated K^+^ channels (*I*_Kv_) or delayed rectifier potassium currents^21^. They are comprised of heteromeric tetramers of four pore-forming α subunits, including three *Kcnb1* subunits and one *Kcnv2* subunit^22^. Similar to *I*_CNG_, *I*_Ca_ is mainly responsible for continuous Ca^2+^ influx into the synaptic terminal. This channel is composed of the pore-forming α_1_ subunit and the auxiliary α_2_δ and β subunits. The *Cacna1f* or Cav1.4 gene encodes the α_1F_-subunit of the voltage-gated L-type Ca^2+^ channel (*I*_CaL_), predominantly expressed in mouse rods^40^. The α_1F_-subunit consists of four homologous domains with six α-helical transmembrane-spanning segments in each domain. *I*_KCa_ and *I*_ClCa_, Ca^2+^-dependent channels, have been identified in photoreceptors. *I*_KCa_ has been reported in the tiger salamander retina^36^, but it remains unclear whether *I*_KCa_ can be found in mouse rods^12,41^. *I*_ClCa_ has a critical physiological function and is widely expressed in many cells. In mouse rods, *I*_ClCa_ regulates signal transmission at synaptic terminals^26^. The electrophysiological characteristics of *I*_ClCa_ are controlled by the membrane voltage and free intracellular Ca^2+^ concentration^9,10^.

To establish and maintain ion homeostasis, additional ionic currents are needed. In the current study, we have added five ionic currents and five unidentified leak currents, including *I*_NaK_, *I*_PMCA_, Na^+^/Ca^2+^ exchanger (*I*_NCX_), the flux of Na^+^–K^+^–2Cl^−^ cotransporter (*J*_NKCC1_), the flux of K^+^–Cl^−^ cotransporter (*J*_KCC2_), outer segment Ca^2+^ leak channel (*I*_L,Caos_), inner segment Ca^2+^ leak channel (*I*_L,Cais_), K^+^ leak channel (*I*_L,K_), Na^+^ leak channel (*I*_L,Na_), and Cl^−^ leak channel (*I*_L,K_). Two types of ATP-dependent pumps are found in mouse rod photoreceptors, *I*_NaK_ and *I*_PMCA_. *I*_NaK_ has been shown to have high expression levels in the inner segment of mouse rods^42,43^. *I*_NaK_ consists of two subunits, α and β subunits. The α_3_β_2_ isoform (*Atp1a3*/*Atp1b2*) is mainly expressed in mouse rods^12^, and Na^+^, K^+^, and ATP binding sites are located at the α_3_-subunit. *I*_NaK_ plays a pivotal role in maintaining the concentration gradients for Na^+^ and K^+^. One ATP molecule is hydrolyzed to pump three Na^+^ ions out and two K^+^ ions into the cell. *I*_PMCA_ has a crucial role in regulating intracellular Ca^2+^ homeostasis. One Ca^2+^ is pumped out for each hydrolyzed ATP via *I*_PMCA_ pumps. The *Atp2b1* isoform of *I*_PMCA_ is highly expressed in the *I*_PMCA_ of mouse rods^12^. *I*_NCX_ extrudes one Ca^2+^ from the cell in exchange for three Na^+^ ions entering the cell. There are three isoforms of *I*_NCX_, including the *Slc8a1*, *Slc8a2, and Slc8a3* isoforms. It remains unclear which isoform is expressed in mouse rods. Johnson et al.^44^ showed high expression of the *Slc8a1* isoform. However, Clark et al.^12^ only found low expression of the *Slc8a3* isoform in mouse rods. Two electroneutral types of cotransporters have been identified in photoreceptors consisting of Na^+^–K^+^–2Cl^−^ and K^+^–Cl^−^ cotransporters^12,45^. The Na^+^–K^+^–2Cl^−^ cotransporter is a major Cl^−^ uptake transporter predominantly expressed in pre- and postsynaptic regions of the outer plexiform layer in the mouse retina^45^. In contrast, the K^+^–Cl^−^ cotransporter is a primary Cl^−^ extruder. Both Na^+^–K^+^–2Cl^−^ and K^+^–Cl^−^ cotransporters are essential for maintaining cell volume and Cl^−^ homeostasis. To date, the role of Cl^−^ in mouse rods remains unclear. Additionally, five unidentified leak channels were necessary for model fitting, consisting of three unidentified leak channels, including K^+^, Na^+^, and Cl^−^, and two unidentified Ca^2+^ leak channels divided by location (outer and inner segments).

### Mathematical model

Earlier mathematical models have been proposed with significant ionic currents^9–11^; however, they have some limitations for maintaining ion homeostasis, which is necessary for estimating energy consumption. In the current study, a novel mathematical model of the mouse rod was developed from our previous works^46^. We introduced mathematical formulas for ionic current activities combined with a phototransduction model. The changes in ionic currents with the alteration in ion concentration were newly defined by the classical Goldman–Hodgkin–Katz (GHK) constant field and Nernst equations, which are widely used for explaining cell membrane electrophysiological phenomena. The membrane voltage of the mouse rod photoreceptor was determined by the ordinary differential equation (Eq. 1).

1 $${C}_{\mathrm{m}}\frac{d{V}_{\mathrm{m}}}{dt}=-{I}_{\mathrm{All}},$$

where ‘*C*_m_’ and ‘*V*_m_’ are the membrane capacitance and potential, respectively. ‘*I*_All_’ is the sum of all ionic currents (Fig. 1, Table 1). The ionic currents ‘*I*’ were calculated by using a combination of available electrophysiological recordings and mathematical formulas similar to those in earlier studies^10,23,47–54^, which can be classified into three groups: (1) single ion flux channel (Eq. 2) for *I*_Kv_, *I*_KCa_, and *I*_ClCa_, (2) multiple ion flux channels (Eq. 3) for *I*_CNG_, *I*_h_, and *I*_CaL_, and (3) simplified formulas from published data for *I*_NCKX_, *I*_PMCA_, *I*_NCX_, *I*_NaK_, *J*_NKCC1_, and *J*_KCC2_ (Supplementary Materials).

2 $$I=g\cdot {m}^{\mathrm{M}}\cdot h\cdot \left({V}_{\mathrm{m}}-{E}_{\mathrm{ion}}\right);\mathrm{ ion }= \{{\mathrm{K}}^{+}, {\mathrm{Cl}}^{-}\}$$

3 $$I=g\cdot {m}^{\mathrm{M}}\cdot h\cdot \sum_{ion}{C}_{F,ion};\mathrm{ ion }= \{{\mathrm{Ca}}^{2+},{\mathrm{ K}}^{+},{\mathrm{ Na}}^{+}\}$$

where the activation variable, inactivation variable, and gating exponent are represented by ‘*m*’, ‘*h*’, and ‘M’, respectively. The modifications and the detailed equations are described in the Supplementary Materials. The ‘*g*’ refers to conductance. The reversal potential (*E*_ion_) of Ca^2+^, K^+^, Na^+^, and Cl^-^ is based on the Nernst equation (Eq. 4), and ‘*C*_F,ion_’ is the GHK constant field equation (Eq. 5).

4 $${E}_{\mathrm{ion}}=\frac{R\cdot T}{z\cdot F\cdot {10}^{-3}}\cdot \mathrm{ln}\left(\frac{{\left[\mathrm{ion}\right]}_{\mathrm{o}}}{{\left[\mathrm{ion}\right]}_{\mathrm{i}}}\right);\mathrm{ ion }= \{{\mathrm{Ca}}^{2+}, {\mathrm{K}}^{+}, {\mathrm{Na}}^{+}, {\mathrm{Cl}}^{-}\}$$

5 $${C}_{\mathrm{F},\mathrm{ion}}=\frac{z\cdot F\cdot {V}_{\mathrm{m}}\cdot {10}^{-3}}{R\cdot T}\cdot \frac{{\left[\mathrm{ion}\right]}_{\mathrm{i}}-{\left[\mathrm{ion}\right]}_{\mathrm{o}}\cdot {e}^{-\frac{z\cdot F\cdot {V}_{\mathrm{m}}\cdot {10}^{-3}}{R\cdot T}}}{1-{e}^{-\frac{z\cdot F\cdot {V}_{\mathrm{m}}\cdot {10}^{-3}}{R\cdot T}}};\mathrm{ ion }= \{{\mathrm{Ca}}^{2+}, {\mathrm{K}}^{+}, {\mathrm{Na}}^{+}\}$$

where ‘*F*’ is Faraday’s constant, ‘*T*’ is the temperature in kelvins, and ‘*R*’ is the ideal gas constant. [ion]_o_ and [ion]_i_ are the concentrations of extracellular and intracellular ion species, respectively. The valence of the ion is ‘*z*’. The calculations of the intracellular calcium systems were developed by using the actual currents and buffering systems from earlier mathematical studies^10,52^. The concentration of Ca^2+^ in outer segments is controlled by a dynamic balance between influx via *I*_CNG_ channels and extrusion via the *I*_NCKX_ exchanger (Eq. 6) (see Supplementary Materials 3 for details). At the inner segment, the calcium influx is organized by the *I*_CaL_ current, and the calcium is released via two ionic currents, *I*_NCX_ and *I*_PMCA_ (Eq. 7).

6 $$\frac{d{\left[{\mathrm{Ca}}^{2+} \right]}_{\mathrm{os}}}{dt}=\frac{-({I}_{\mathrm{CNG},\mathrm{Ca}}-2\cdot {I}_{\mathrm{NCKX}}+{I}_{\mathrm{L},\mathrm{Caos}})}{2\cdot F\cdot {V}_{\mathrm{os}}}-\frac{d{\left[{\mathrm{Ca}}^{2+}\right]}_{\mathrm{os},\mathrm{b}}}{dt}-\frac{{J}_{\mathrm{dif}}}{{V}_{\mathrm{os}}}$$

7 $$\frac{d{\left[{\mathrm{Ca}}^{2+}\right]}_{\mathrm{is}}}{dt}=\frac{-({I}_{\mathrm{CaL},\mathrm{Ca}}+{I}_{\mathrm{PMCA}}-2\cdot {I}_{\mathrm{NCX}}+{I}_{\mathrm{L},\mathrm{Cais}})}{2\cdot F\cdot {V}_{\mathrm{is}}}-\frac{d{\left[{\mathrm{Ca}}^{2+}\right]}_{\mathrm{is},\mathrm{b}}}{dt}+\frac{{J}_{\mathrm{dif}}}{{V}_{\mathrm{is}}}$$

where ‘[Ca^2+^]’ is the calcium concentration, ‘*V*’ is the cell volume, and subscripts ‘os’, ‘is’, ‘b’, and ‘dif’ refer to the outer segment, inner segment, buffer, and diffusion, respectively. The changes in each [ion]_i_ were estimated by the flow of individual ionic currents and given by $d\left[\mathrm{ion}\right]/dt=I/(z\cdot F\cdot V)$. The energy expenditure from ionic current activities was estimated by the flows of Na^+^ uptake via *I*_CNG_, *I*_NCKX_, *I*_h_, *I*_CaL_, *I*_NCX_, *J*_NKCC1_, and *I*_L,Na_. Every ATP molecule is hydrolyzed to transport three Na^+^ out of the cell via *I*_NaK_ (Eq. 8). Ca^2+^ uptake and release were described by Eq. (7); however, one ATP molecule is required for the extrusion of excess Ca^2+^ via *I*_PMCA,_ and the required ATP was determined by Eq. (9) (see Supplementary Materials for *d*[Na^+^]_influx_/*dt* & *d*[Ca^2+^]_influx_/*dt*).

8 $${\text{ATP}}\,{\text{for}}\,{\text{pumping}}\,{\text{out}}\,{\text{excess}}\,{\text{Na}}^{ + } = \frac{{d\left[ {{\text{Na}}^{ + } } \right]_{{{\text{influx}}}} }}{dt} \cdot \frac{{N_{{\text{A}}} \cdot V_{{{\text{cell}}}} }}{3} \cdot 10^{ - 3}$$

9 $${\text{ATP}}\,{\text{for}}\,{\text{pumping}}\,{\text{out}}\,{\text{excess}}\,{\text{Ca}}^{2 + } = \frac{{d\left[ {{\text{Ca}}^{2 + } } \right]_{{{\text{influx}}}} }}{dt} \cdot N_{{\text{A}}} \cdot V_{{{\text{is}}}} \cdot 10^{ - 6}$$

where *N*_A_ refers to the Avogadro constant. In the light environment, additional energy is required for the complex mechanism of the phototransduction cascade. The molecular mechanisms underlying the complex transduction cascade can be described by using the mathematical model from earlier studies^50–54^.

### Time-integration and units

The model parameters of each ionic current were newly fitted with experimental findings and theoretical work. The model was based on the standard differential equation form, and numerical integration was performed by Euler’s method.

The following dimensions were applied in this model: millivolt (mV) for membrane potential, picoampere (pA), picoampere per picofarad (pA/pF) or femtoampere per picofarad (fA/pF) for ionic current, millimolar (mM) or micromolar (μM) for concentration, and second (s) and millisecond (ms) for time. All codes for the simulation program were prepared using Microsoft Visual Studio Community 2019 (Microsoft Corp.).

## Results

### Electrophysiological features at different light-flash intensities

**Figure 2:**
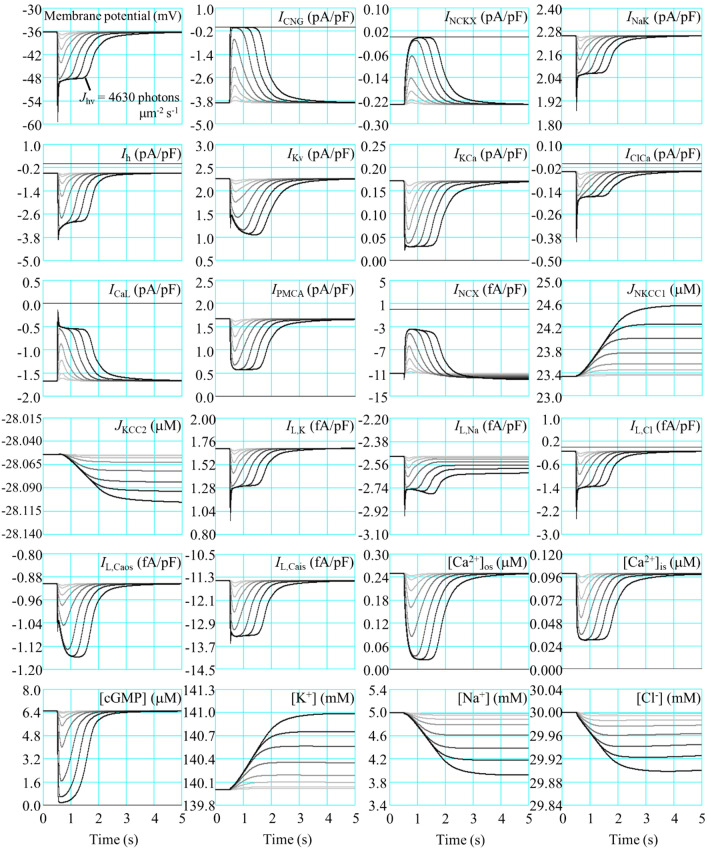
Electrophysiological characteristics of membrane potential and a variety of ionic currents of the mouse rod photoreceptor in response to light-flash exposure. Simulation results of light responses were performed at a variety of light-flash intensities (*J*_hv_); 1.7, 4.8, 15.2, 39.4, 125, 444, 1406, and 4630 photons μm^−2^ s^−1^ from gray to black (with color gradient), respectively. The responses started at time 0.5 s, and the stimuli consisted of 20-ms flashes. The collecting area was 0.43 μm^2^^52^, and the membrane capacitance was 3.6 pF^55^.

**Figure 3:**
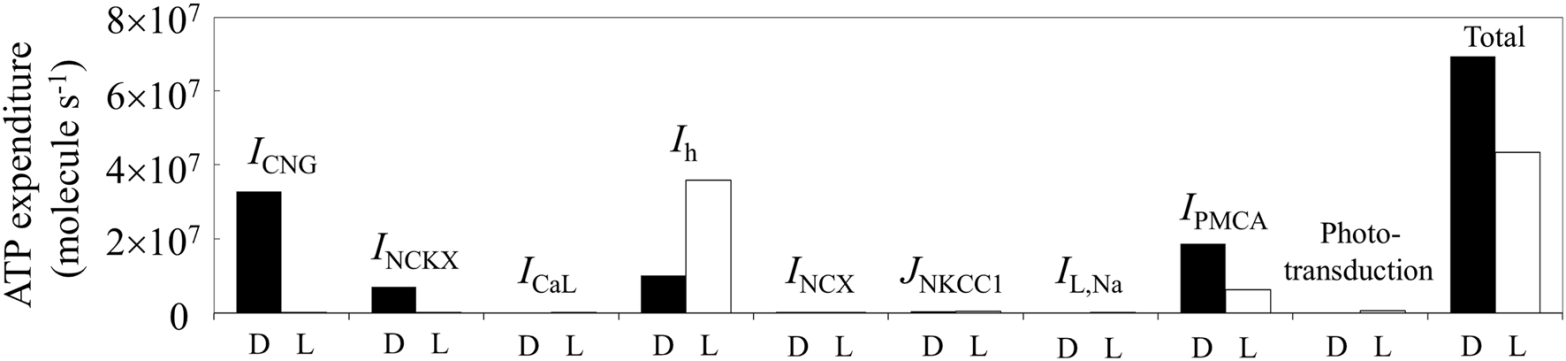
ATP expenditure of mouse rod photoreceptors required for ion extrusion and phototransduction in darkness (D) and in light (L).

When the photoreceptor is exposed to light, the transduction cascades are activated, leading to the closing of the light-sensitive channels and subsequently producing hyperpolarization. In the current study, we reproduced mouse rod photoreceptor responses to various light intensities described in earlier studies^13,52^. Figure 2 shows the simulation results of the mouse rod photoreceptor response to a variety of light-flash intensities. At high light-flash intensities, the membrane voltage was hyperpolarized to below − 55 mV, and *I*_CNG_ and *I*_NCKX_ were close to zero. The amount of light-activated cGMP-phosphodiesterase required to produce a half-maximal response was approximately 43.5 photons μm^−2^ s^−1^, and the time to peak was 185 ms (Fig. 1S). Calcium homeostasis in the outer segment is regulated by a dynamic equilibrium between Ca^2+^ influx and Ca^2+^ extrusion via *I*_CNG_ and *I*_NCKX_, respectively. Hyperpolarization induces the reduction in ion transport events in most ionic currents, except *I*_h_ and *I*_ClCa_. Both *I*_h_ and *I*_ClCa_ were activated in response to light exposure, and their maximum amplitudes were − 3.9 and − 0.4 pA/pF at a time to peak at 47 and 34 ms, respectively. The reduced ion transport activities led to increased [K^+^] and decreased [Ca^2+^], [Na^+^], [Cl^−^], and [cGMP]. However, K^+^, Na^+^, and Cl^−^ concentrations required approximately 785, 723, and 680 s, respectively, to return to their initial value in darkness after high-intensity light-flash simulations. The peak of total energy expenditure via *I*_NaK_, *I*_PMCA_, and phototransduction cascade mechanisms was approximately 7 $\times$ 10^7^ molecules of ATP s^−1^ (Fig. 3).

### Energy expenditure at different constant light intensities

**Figure 4:**
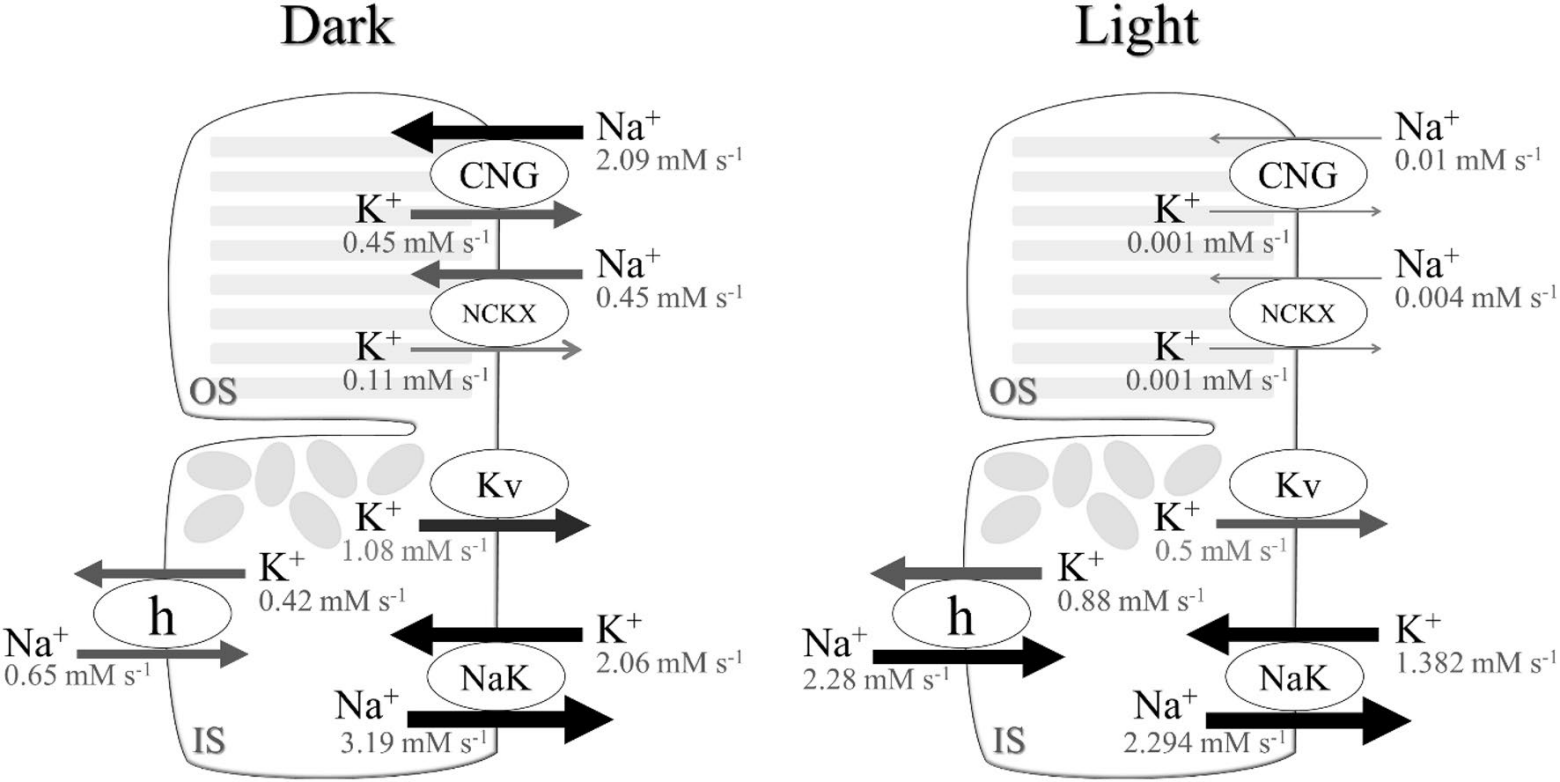
Changes in Na^+^ and K^+^ flow of major ionic currents across the plasma membrane in the current model of mouse rod photoreceptor in darkness and in light. *OS* outer segment, *IS* inner segment with cell body and synaptic terminal.

The total ATP expenditure required for Na^+^ and Ca^2+^ extrusions was 7 $\times$ 10^7^ molecules of ATP s^−1^ in darkness for maintaining ion homeostasis, which significantly decreased after light exposure. Approximately 4.3 $\times$ 10^7^ molecules of ATP s^−1^ (62.4% of total energy expenditure in darkness) were required for ion extrusion and phototransduction under continuous high-intensity light simulation (Fig. 3). An extrusion of excess Na^+^ via *I*_NaK_ consumed 5 $\times$ 10^7^ molecules of ATP s^−1^ in darkness. *I*_CNG_ (65%), *I*_NCKX_ (14%), and *I*_h_ (20%) had significant contributions to Na^+^ uptake in darkness (Fig. 4); the other ion channels (1%), including *I*_CaL_, *I*_NCX_, *J*_NKCC1_, and *I*_L,Na_, however, had no significant impact on the total Na^+^ uptake (Fig. 3). In light, the proportion of total energy expenditure via *I*_h_ increased to 83%, whereas Na^+^ uptake via other currents was close to zero. On the other hand, approximately 2 $\times$ 10^7^ molecules of ATP s^−1^ were consumed for releasing excess Ca^2+^ ions via *I*_PMCA_ in darkness, but only 6.3 $\times$ 10^6^ molecules of ATP s^−1^ were consumed in bright light.

**Figure 5:**
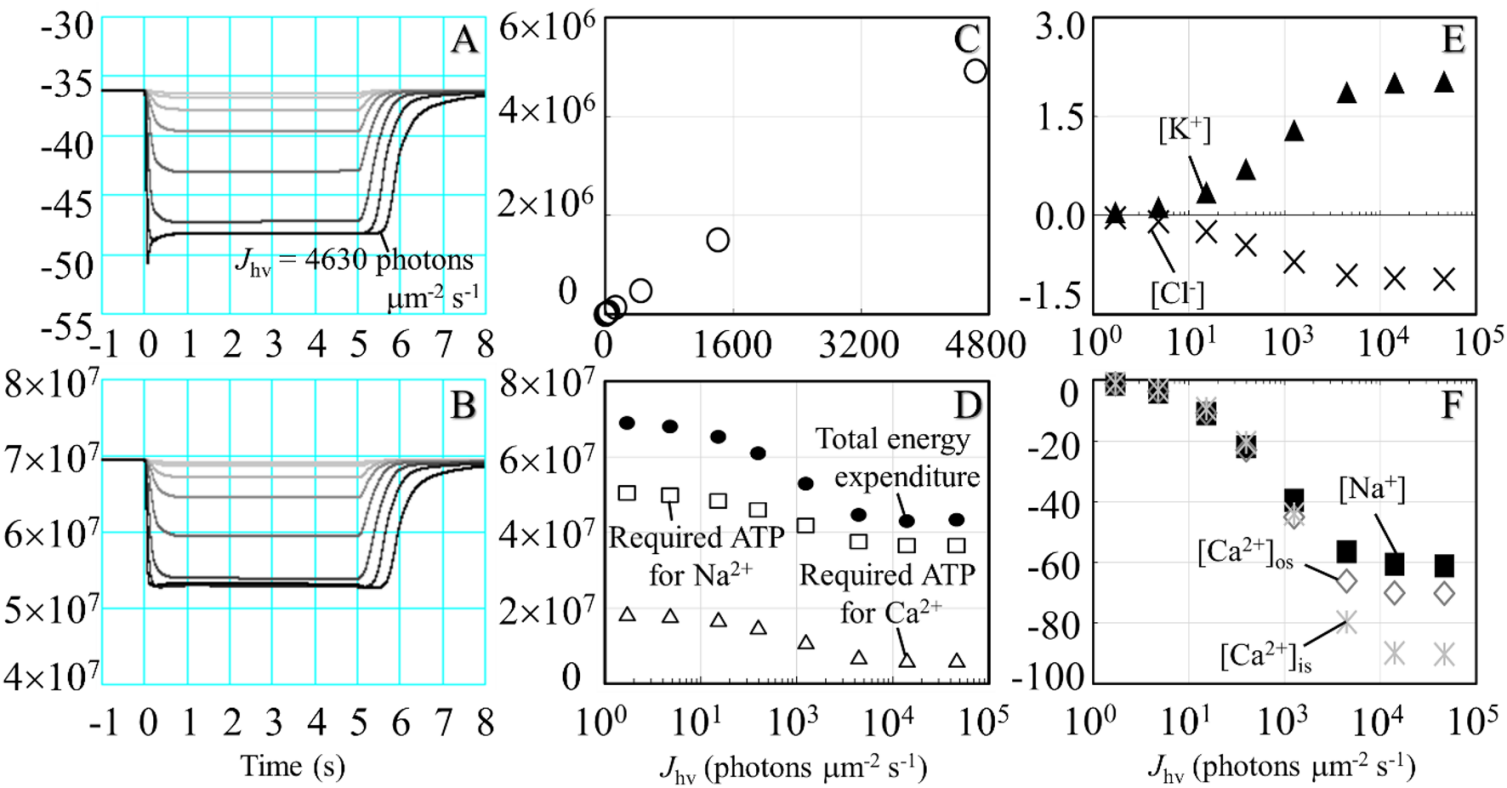
Membrane voltage, energy expenditure, and alterations in ion concentrations during constant light simulations. Simulations were performed at a variety of light intensities (*J*_hv_): 1.7, 4.8, 15.2, 39.4, 125, 444, 1406, and 4630 photons μm^−2^ s^−1^ from gray to black (with color gradient). The responses started at time 0 s, and the stimuli consisted of 5 s of constant light. (**A**) membrane potential (mV), (**B**) total energy expenditure (molecules ATP), (**C**) energy consumption via phototransduction cascades (molecules s^−1^), (**D**) total energy expenditure and required ATP for Na^+^ and Ca^2+^ extrusions (molecules s^−1^), (**E**) alterations of [K^+^] and [Cl^−^] from initial values (% s^−1^), (**F**) alterations of [Na^+^], [Ca^2+^]_os_, and [Ca^2+^]_is_ from initial values (% s^−1^), and (**C**–**F**) each parameter was recorded at 5 s.

The simulation of the mouse photoreceptor in response to 5 s of constant light showed a reduction in membrane potential amplitude. Nevertheless, the plateau phase remained in the range of − 45 to − 50 mV in the high-intensity light-flash simulations (Fig. 5A). The total ATP consumption significantly decreased after light exposure (Fig. 5B). In contrast, the energy consumption via the phototransduction cascade increased linearly with increasing intensity (Fig. 5C). Under high-intensity constant light, the ATP required for the extrusion of Na^+^ and Ca^2+^ decreased by approximately 27.6% and 65.9%, respectively, from the initial value in darkness (Fig. 5D). The levels of K^+^ and Cl^−^ concentrations slightly changed, whereas Na^+^ and Ca^2+^ dramatically decreased under high-intensity constant light simulations (Fig. 5E,F).

## Discussion

At the outer segment, the total current composed of *I*_CNG_ (94.4%) and *I*_NCKX_ (5.6%) was approximately – 15 pA under dark conditions, which has been shown through experimental observations^56,57^. It utilized approximately 4 × 10^7^ molecules of ATP s^−1^ to extrude Na^+^ via *I*_NaK_ (3.3 × 10^7^ molecules of ATP s^−1^ and 7 × 10^6^ molecules of ATP s ^−1^ via *I*_CNG_ and *I*_NCKX_, respectively). After bright light exposure, it fell to nearly zero by approximately 2.7 × 10^6^ ATP s^−1^ per pA of ATP consumption rate. These results are consistent with earlier assumptions^1^, and the electrophysiological characteristics fit well with the experimental data^13^ and mathematical model^52^. The Na^+^ influx of *I*_CNG_ (90% of total *I*_CNG_ influxes) was larger than the Ca^2+^ influx in darkness. The rising phase and the intensity-response curve of *I*_CNG_ were in good agreement with experimental data and theoretical work^50–54^.

*I*_h_ is responsible for shaping the membrane potential response to light, i.e., the characteristics of peak-plateau sag^36^. We modified the activation and inactivation parameters of *I*_h_ to fit with the peak-plateau sag of the membrane potential as previously reported^9–11,17–19^. The hyperpolarized peak membrane potential was below − 55 mV, and the plateau phase was in the range of − 45 to − 50 mV in high-intensity light simulations. *I*_h_ contributed to Na^+^ uptake of approximately 20% in darkness, and Na^+^ uptake increased 3.5 times more than in darkness under continuous high-intensity light simulations. Approximately 3.6 × 10^7^ molecules of ATP s^−1^ were consumed for the extrusion of excess Na^+^ from *I*_h_ in bright light. These results differ from earlier reports^1^ because of a difference in the shape of the characteristic peak-plateau sag of the membrane potential. Energy consumption for the extrusion of excess Ca^2+^ via *I*_PMCA_ in darkness was similar to that reported by Okawa et al.^1^, contributing approximately one-third of the total energy expenditure or half of the energy consumed via dark current in darkness. The model was fitted to maintain intracellular Ca^2+^ homeostasis via *I*_PMCA_ and *I*_NCX_ for Ca^2+^ extrusion and *I*_CaL_ and *I*_L,Cais_ for Ca^2+^ uptake. However, the roles of Ca^2+^ extrusion via *I*_PMCA_ and *I*_NCX_ in mouse rod photoreceptors are not fully understood. Immunohistochemically, mammalian photoreceptor synaptic terminals have revealed strong and very weak staining for *I*_PMCA_ and *I*_NCX_, respectively^58^. It was suggested that *I*_CaL_ and *I*_PMCA_ play significant roles in maintaining intracellular Ca^2+^ homeostasis in mammalian photoreceptors. The *I*_CaL_ is responsible for the neurotransmission of visual signals. Mutations in the human Cav1.4 gene encoding *I*_CaL_ have been associated with congenital stationary night blindness type 2. This disease has been functionally classified into three types: loss-of-function, gain-of-function, and C-terminal modulator function impairment^59^, which may lead to an increase or decrease in energy consumption for Ca^2+^ extrusion via *I*_PMCA_. However, whether there is an increase or decrease in *I*_PMCA_ density has not yet been shown in any of the mutations in Cav1.4 genes.

*I*_NaK_ plays critical roles in K^+^ uptake and Na^+^ extrusion to maintain a high level of K^+^ and a low level of Na^+^ concentrations in photoreceptors to establish the normal resting membrane potential. To date, *I*_NaK_ is the only pump that can release excess Na^+^ ions. The α_3_β_2_ isoform current-membrane potential relationship was characterized by the voltage-dependent current using the Boltzmann relation^23,60^. It can operate at approximately 80% and 65% of the saturated maximum of *I*_NaK_ at resting and *V*_m_ less than − 55 mV, respectively. The current model demonstrated a fast recovery of *I*_NaK_ activity after photoreceptor hyperpolarization, resulting in decreased Na^+^ and increased K^+^. However, the transitional state of *I*_NaK_ from darkness to light remains unresolved. Further experimental studies may need to consider the difference in ion concentration between darkness and light. An inability to sustain ion homeostasis, such as ATP depletion or ion pump failure, can lead to apoptosis and pathogenesis^61^. The excess K^+^ was mainly released from photoreceptors via *I*_Kv_, *I*_CNG_, and *I*_h_, with approximately 49.5%, 20.9%, and 19.3% of total K^+^ extrusion in darkness and 35.1%, 0%, and 61.8% of absolute K^+^ extrusion under continuous high-intensity light simulations, respectively (Fig. 4 and Table 2). The contributions of the ionic channels to the outward current in darkness were 35.5%, 35.5%, 26.2%, and 3% for *I*_Kv_,* I*_NaK_,* I*_PMCA_, and *I*_KCa_, respectively, which are similar to earlier assumptions^23^. However, *I*_KCa_ channels are expressed at a low level, as shown in the gene expression database of mouse rods^12^.

**Table 2:** Percentage of total K^+^ extrusion in photoreceptors via principal ion currents.

Ion currents	Darkness (%)	Light (%)
*I*_Kv_	49.5	35.1
*I*_CNG_	20.9	0
*I*_h_	19.3	61.8

**Figure 6:**
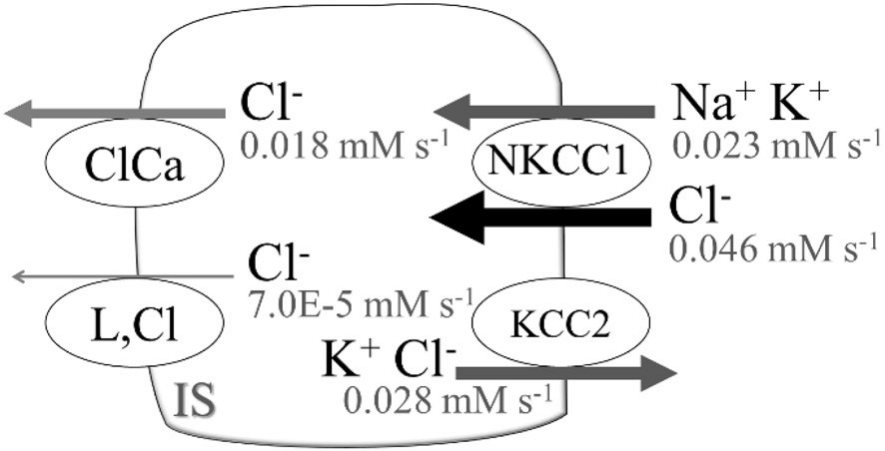
Chloride homeostasis in the mouse rod in the current study. *IS* inner segment with cell body and synaptic terminal.

*I*_ClCa_, *I*_NKCC1_, *I*_KCC2_, and *I*_L,Cl_ associated with chloride fluxes play important roles in stabilizing the membrane potential of photoreceptors during presynaptic activity and presynaptic Ca^2+^ channel modulation. Nevertheless, changes in intracellular Cl^−^ concentration within the physiological range are not fully understood. Note that in the current study, chloride influx and efflux were adjusted to maintain the low energy requirement for Na^+^ extrusion. Using the mathematical formulas for *I*_ClCa_^9,10^, the simulation results of the electrophysiological characteristics showed different features due to the effects of the electrochemical driving force manifested by the Nernst equation. On the other hand, two electroneutral types of cation-chloride cotransporters, *I*_NKCC1_ and *I*_KCC2_, were crucial for cellular homeostasis (Fig. 6). *I*_NKCC1_ is strongly expressed at rod terminals. This cotransporter is responsible for Cl^−^ uptake, maintaining a Cl^−^ equilibrium potential. Unlike *I*_NKCC1_, *I*_KCC2_ is the main Cl^−^ extruder that promotes fast hyperpolarizing postsynaptic inhibition in the brain^62^ and inner retina^45^, but the role of *I*_KCC2_ in mouse rods is still unclear. Several simple mathematical equations exist to evaluate chloride regulation in neurons^49,63^. These equations can elucidate the depolarizing effect of *I*_NKCC1_ and the hyperpolarizing effects of *I*_KCC2_, a concept widely acknowledged in the field^64^. However, the mathematical formulations for mouse rods remain poorly understood.

After high-intensity light-flash simulations, the amounts of intracellular K^+^, Na^+^, and Cl^−^ rapidly changed; however, the times of return of K^+^, Na^+^, and Cl^−^ to their initial values were approximately 700 s, which subsequently prolonged energy balancing times. The changes in the intracellular concentration showed no significant difference in reversal potential, and thus they did not affect ionic currents. Nevertheless, whether K^+^, Na^+^, and Cl^−^ ion accumulation or depletion causes retinal disease in mouse rods is unclear.

This study focuses on the utilization of mathematical formulas to evaluate the activities of ionic currents and factors governing ATP consumption within mouse rod photoreceptors under both darkness and light exposure. The model effectively mirrors physiological ranges, thereby closely aligning itself with empirical observations. Based on the findings derived from controlled physiological experiments, the current proposed model narrows its focuses to the short-term regulatory mechanisms governing cell status. However, it does not encompass the broader context of long-term cellular remodeling processes. Future investigations will delve into exploring specific transitional states, particularly those involving shifts from physiological to pathophysiological conditions. This exploration will encompass the effects of mitochondrial dysfunction, hypoxia, and gene mutations on energy utilization. Conversely, a comprehensive scrutiny of the energy expenditure of cone photoreceptors, recognized for their elevated energy requirements compared to rods under both dark and light conditions^17^, holds the potential to yield profound insights into the fundamental distinctions between the energy regulatory mechanisms of rods and cones.

Furthermore, the current model can provide a practical framework for future clinical and experimental studies for a better understanding of the pathophysiology of the retina and improve potential future treatments. However, further research on coupling the photoreceptor model with experiments is urgently needed to fill the scientific gaps in the complex phenomenon and increase the model’s accuracy and specificity.

### Supplementary Information

Supplementary Information.

## Data Availability

All data generated or analyzed during this study are included in this published article [and its supplementary information files]. The datasets used and/or analyzed during the current study available from the corresponding author on reasonable request.
