# Supplementary material for: Clarifying the composition of the ATP consumption factors required for maintaining ion homeostasis in mouse rod photoreceptors
Source: Sci Rep. 2023 Aug 29;13:14161. doi: 10.1038/s41598-023-40663-y (PMC10465610; doi:10.1038/s41598-023-40663-y)
Supplement: Supplementary file 1 — Supplementary Information. [file 41598_2023_40663_MOESM1_ESM.docx]

**Supplementary Materials**

**Clarifying the composition of the ATP consumption factors required for maintaining ion homeostasis in mouse rod photoreceptors**

Muangkram Yuttamol^1^*, Himeno Yukiko^1^, and Amano Akira^1^

^1^Department of Bioinformatics, College of Life Sciences, Ritsumeikan University

*Corresponding Author

**Contents**

- **S1** Summary of model modifications
- **S2** Intensity-response relationship
- **S3** Mathematical description of the mouse rod photoreceptor

**S1 Summary of model modifications**

**Table 1S** The modifications of the current model equations

| Parameters | References;  original equations | Modification |
| --- | --- | --- |
| **Outer segment** |  |  |
| Phototransduction | Hamer et al. 2005^a^ | - |
| Ca^2+^ dynamics | Hamer et al. 2005^a^ | Unidentified Ca^2+^ leak channel and Ca^2+^ diffusion from outer to inner segments |
| *I*_CNG_ | Hamer et al. 2005^a^ | Adding GHK equations |
| *I*_NCKX_ | Hamer et al. 2005^a^ | - |
| **Inner segment** |  |  |
| Ca^2+^ dynamics | Kamiyama et al. 2009 | Detailed Ca^2+^ influx and efflux mechanisms, and Ca^2+^ diffusion from outer to inner segments |
| *I*_CaL_ | Current study | Adding GHK equations and model fitting |
| *I*_h_ | Kamiyama et al. 2009 | Adding GHK equations and model fitting |
| *I*_Kv_ | Fortenbach et al. 2021 | - |
| *I*_KCa_ | Kamiyama et al. 2009 | Adding Nernst equation and model fitting |
| *I*_ClCa_ | Kamiyama et al. 2009 | Adding Nernst equation |
| *I*_NaK_ | Fortenbach et al 2021 | Model fitting |
| *I*_PMCA_ | Takeuchi et al. 2006 and  Kamiyama et al. 2009 | Model fitting |
| *I*_NCX_ | Luo and Rudy 1994 | Model fitting |
| *I*_NKCC1_ | Wei et al. 2014 | Model fitting |
| *I*_KCC2_ | Wei et al. 2014 | Model fitting |
| **New parameters** | Current study | Estimating the alterations of K^+^, Na^+^, and Cl^-^ concentrations  Estimating the amount of energy consumptions via ionic currents, and phototransduction in darkness and in light  Unidentified K^+^, Na^+^, and Cl^-^ leak currents |

^a^Hamer et al. 2005, Dell'Orco et al. 2009, Invergo et al. 2014, Dell'Orco and Dal Cortivo 2019, Beelen et al. 2021

*Noted* that Model fitting is based on experimental mouse data and theoretical work (Table 1 in the main text), and GHK Eqs. are the classical Goldman–Hodgkin–Katz constant field equations.

**S2 Intensity-response relationship**

The relationship between the peak amplitude of *I*_CNG_ and the levels of light intensities is illustrated in Fig. S1 (*see* Dell'Orco et al. 2009, Invergo et al. 2014, Dell'Orco and Dal Cortivo 2019, Beelen et al.

2021).


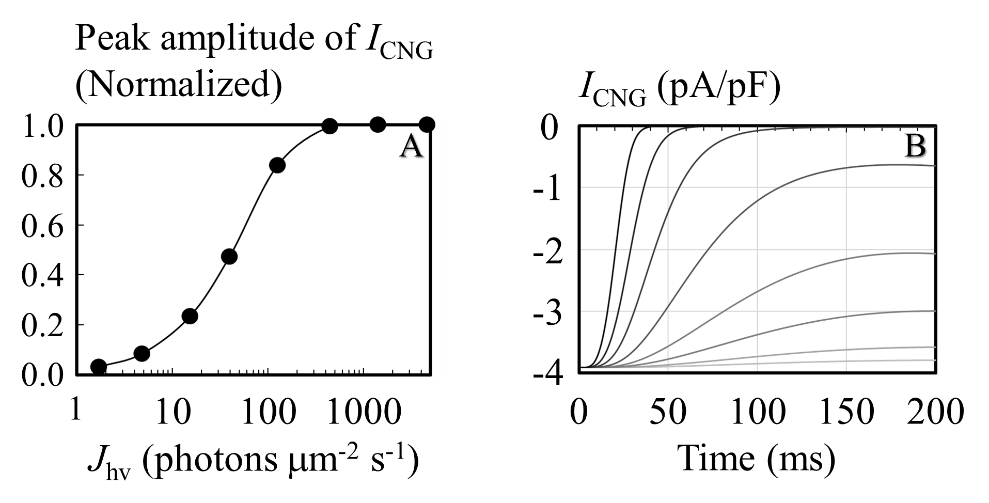


**Fig. 1S** A sigmoidal shape showing hyperpolarization of the membrane potential with the increased light-flash intensities (*J*_hv_) and rising phase of *I*_CNG_. Simulation results of light responses were performed in a variety of *J*_hv_; 1.7, 4.8, 15.2, 39.4, 125, 444, 1406, and 4630 photons μm^-2^ s^-1^. A) exponential saturation relationship between the peak amplitude of *I*_CNG_ and a variety of light intensities (filled circle), B) rising phase of photocurrent response to light-flash simulations in grey to black (with color gradient). The responses started at time 0 s, and the stimuli consisted of 20 ms flashes.

**S3 Mathematical description of the mouse rod photoreceptor**

**Membrane potential (*V*_m_ [mV])**

$$I_{\mathrm{All}}=I_{\mathrm{CNG}}+I_{\mathrm{NCKX}}+I_{\mathrm{Kv}}+I_{\mathrm{KCa}}+I_{h}+I_{\mathrm{ClCa}}+I_{\mathrm{NaK}}+I_{\mathrm{NCX}}+{I_{\mathrm{CaL}}+I}_{\mathrm{PMCA}}+I_{L,\mathrm{Caos}}+I_{L,\mathrm{Cais}}+I_{L,K}+I_{L,\mathrm{Na}}+I_{L,\mathrm{Cl}}$$

$$C_{m}\frac{dV_{m}}{dt}=-I_{\mathrm{All}} (V_{m}\left( 0 \right)=-36.186)$$

*C*_m_ = 3.6 pF (Ingram et al. 2019); *Note* that *J*_NKCC1_ and *J*_KCC2_ are electroneutral.

**Reversal potential (*E*_ion_) based on Nernst equation and the classical Goldman–Hodgkin–Katz constant field equation (*C*_F_)**

$$E_{K}=\frac{R\cdot T}{1\cdot F\cdot{10}^{-3}}\cdot\ln\left( \frac{\left[ K^{+} \right]_{o}}{\left[ K^{+} \right]_{i}} \right)$$

$$E_{\mathrm{Na}}=\frac{R\cdot T}{1\cdot F\cdot{10}^{-3}}\cdot\ln\left( \frac{\left[ \mathrm{Na}^{+} \right]_{o}}{\left[ \mathrm{Na}^{+} \right]_{i}} \right)$$

$$E_{\mathrm{Cl}}=\frac{R\cdot T}{-1\cdot F\cdot{10}^{-3}}\cdot\ln\left( \frac{\left[ \mathrm{Cl}^{-} \right]_{o}}{\left[ \mathrm{Cl}^{-} \right]_{i}} \right)$$

$$E_{\mathrm{Cais}}=\frac{R\cdot T}{2\cdot F\cdot{10}^{-3}}\cdot\ln\left( \frac{\left[ \mathrm{Ca}^{2+} \right]_{o}}{\left[ \mathrm{Ca}^{2+} \right]_{\mathrm{is}}} \right)$$

$$E_{\mathrm{Caos}}=\frac{R\cdot T}{2\cdot F\cdot{10}^{-3}}\cdot\ln\left( \frac{\left[ \mathrm{Ca}^{2+} \right]_{o}}{\left[ \mathrm{Ca}^{2+} \right]_{\mathrm{os}}} \right)$$

$$C_{F,K}=\frac{1\cdot F\cdot V_{m}\cdot{10}^{-3}}{R\cdot T}\cdot\frac{\left( \left[ K^{+} \right]_{i}-\left[ K^{+} \right]_{o}\cdot\exp\left( \frac{-1\cdot F\cdot V_{m}\cdot{10}^{-3}}{R\cdot T} \right) \right)}{\left( 1-\exp\left( \frac{-1\cdot F\cdot V_{m}\cdot{10}^{-3}}{R\cdot T} \right) \right)}$$

$$C_{F,Na}=\frac{1\cdot F\cdot V_{m}\cdot{10}^{-3}}{R\cdot T}\cdot\frac{\left( \left[ \mathrm{Na}^{+} \right]_{i}-\left[ \mathrm{Na}^{+} \right]_{o}\cdot\exp\left( \frac{-1\cdot F\cdot V_{m}\cdot{10}^{-3}}{R\cdot T} \right) \right)}{\left( 1-\exp\left( \frac{-1\cdot F\cdot V_{m}\cdot{10}^{-3}}{R\cdot T} \right) \right)}$$

$$C_{F,Cais}=\frac{2\cdot F\cdot V_{m}\cdot{10}^{-3}}{R\cdot T}\cdot\frac{\left( \left[ \mathrm{Ca}^{2+} \right]_{\mathrm{is}}\cdot{10}^{-3}-\left[ \mathrm{Ca}^{2+} \right]_{o}\cdot{10}^{-3}\cdot\exp\left( \frac{-2\cdot F\cdot V_{m}\cdot{10}^{-3}}{R\cdot T} \right) \right)}{\left( 1-\exp\left( \frac{-2\cdot F\cdot V_{m}\cdot{10}^{-3}}{R\cdot T} \right) \right)}$$

$$C_{F,Caos}=\frac{2\cdot F\cdot V_{m}\cdot{10}^{-3}}{R\cdot T}\cdot\frac{\left( \left[ \mathrm{Ca}^{2+} \right]_{\mathrm{os}}\cdot{10}^{-3}-\left[ \mathrm{Ca}^{2+} \right]_{o}\cdot{10}^{-3}\cdot\exp\left( \frac{-2\cdot F\cdot V_{m}\cdot{10}^{-3}}{R\cdot T} \right) \right)}{\left( 1-\exp\left( \frac{-2\cdot F\cdot V_{m}\cdot{10}^{-3}}{R\cdot T} \right) \right)}$$

*F* = 96485.34 C mol^-1^, *R* = 8.3145 J mol^-1^ K^-1^, *T* = 310 K, [K^+^]_o_ = 5 mM, [K^+^]_i_ = 140 mM, [Na^+^]_o_ = 145 mM, [Na^+^]_i_ = 5 mM, [Cl^-^]_o_ = 110 mM, [Cl^-^]_i_ = 30 mM, [Ca^2+^]_o_ = 1600 μM, [Ca^2+^]_os_ = 0.25 μM, [Ca^2+^]_is_ = 0.1 μM; *Note* that the concentrations are based on the typical mammalian neurons.

**Calcium system [μM] at outer segment**

Model equations; Dell'Orco et al. 2009, Invergo et al. 2014, Dell'Orco and Dal Cortivo 2019, Beelen et al. 2021

$$\frac{d\left[ \mathrm{Ca}^{2+} \right]_{\mathrm{os}}}{dt}=\frac{-(I_{CNG,Ca}-2\cdot I_{\mathrm{NCKX}}+I_{L,Caos})}{2\cdot F\cdot V_{\mathrm{os}}}\cdot{10}^{-6}-\frac{d\left[ \mathrm{Ca}^{2+} \right]_{os,b}}{dt}-\frac{J_{\mathrm{dif}}}{V_{\mathrm{os}}}$$

$\left[ \mathrm{Ca}^{2+} \right]_{os,b}$ is the concentration of buffered calcium.

$$\frac{d\left[ \mathrm{Ca}^{2+} \right]_{os,b}}{dt}=k_{1}\cdot\left( e_{T}-\left[ \mathrm{Ca}^{2+} \right]_{os,b} \right)\cdot\left[ \mathrm{Ca}^{2+} \right]_{\mathrm{os}}-k_{2}\cdot\left[ \mathrm{Ca}^{2+} \right]_{os,b}$$

The $J_{\mathrm{dif}}$ is the rate of Ca^2+^ diffusion between outer and inner segments.

$$J_{\mathrm{dif}}=k_{3}\cdot\left( \left[ \mathrm{Ca}^{2+} \right]_{\mathrm{os}}-\left[ \mathrm{Ca}^{2+} \right]_{\mathrm{is}} \right)$$

*k*_1_ = 9.37059 s^-1^ μM^-1^, *k*_2_ = 46.412 s^-1^, *k*_3_ = 1$\times$10^-20^ s^-1^ μM^-1^, *e*_T_ = 400 μM, *V*_os_ = 3.916$\times$10^-14^ L

**Calcium system [μM] at inner segment**

Model equations; Kamiyama et al. 2009

$$\frac{d\left[ \mathrm{Ca}^{2+} \right]_{\mathrm{is}}}{dt}=\frac{-(I_{\mathrm{CaL},\mathrm{Ca}}+I_{\mathrm{PMCA}}-2\cdot I_{\mathrm{NCX}}+I_{L,Cais})}{2\cdot F\cdot V_{\mathrm{is}}}\cdot{10}^{-6}-\frac{D_{\mathrm{Ca}}\cdot S_{1}}{\delta\cdot V_{\mathrm{is}}}\cdot\left( \left[ \mathrm{Ca}^{2+} \right]_{\mathrm{is}}-\left[ \mathrm{Ca}^{2+} \right]_{\mathrm{if}} \right)-\frac{d\left[ \mathrm{Ca}^{2+} \right]_{\mathrm{ls}}}{dt}-\frac{d\left[ \mathrm{Ca}^{2+} \right]_{\mathrm{hs}}}{dt}+\frac{J_{\mathrm{dif}}}{V_{\mathrm{is}}}$$

$$\frac{d\left[ \mathrm{Ca}^{2+} \right]_{\mathrm{if}}}{dt}=\frac{D_{\mathrm{Ca}}\cdot S_{1}}{\delta\cdot V_{\mathrm{if}}}\cdot\left( \left[ \mathrm{Ca}^{2+} \right]_{\mathrm{is}}-\left[ \mathrm{Ca}^{2+} \right]_{\mathrm{if}} \right)-\frac{d\left[ \mathrm{Ca}^{2+} \right]_{\mathrm{lf}}}{dt}-\frac{d\left[ \mathrm{Ca}^{2+} \right]_{\mathrm{hf}}}{dt} (\left[ \mathrm{Ca}^{2+} \right]_{\mathrm{if}}(0)=0.1)$$

$$\frac{d\left[ \mathrm{Ca}^{2+} \right]_{\mathrm{ls}}}{dt}=L_{b1}\cdot\left[ \mathrm{Ca}^{2+} \right]_{\mathrm{is}}\cdot\left( B_{L}-\left[ \mathrm{Ca}^{2+} \right]_{\mathrm{ls}} \right)-L_{b2}\cdot\left[ \mathrm{Ca}^{2+} \right]_{\mathrm{ls}} (\left[ \mathrm{Ca}^{2+} \right]_{\mathrm{ls}}\left( 0 \right)=80)$$

$$\frac{d\left[ \mathrm{Ca}^{2+} \right]_{\mathrm{hs}}}{dt}=H_{b1}\cdot\left[ \mathrm{Ca}^{2+} \right]_{\mathrm{is}}\cdot\left( B_{H}-\left[ \mathrm{Ca}^{2+} \right]_{\mathrm{hs}} \right)-H_{b2}\cdot\left[ \mathrm{Ca}^{2+} \right]_{\mathrm{hs}} (\left[ \mathrm{Ca}^{2+} \right]_{\mathrm{hs}}\left( 0 \right)=30)$$

$$\frac{d\left[ \mathrm{Ca}^{2+} \right]_{\mathrm{lf}}}{dt}=L_{b1}\cdot\left[ \mathrm{Ca}^{2+} \right]_{\mathrm{if}}\cdot\left( B_{L}-\left[ \mathrm{Ca}^{2+} \right]_{\mathrm{lf}} \right)-L_{b2}\cdot\left[ \mathrm{Ca}^{2+} \right]_{\mathrm{lf}} (\left[ \mathrm{Ca}^{2+} \right]_{\mathrm{lf}}\left( 0 \right)=80)$$

$$\frac{d\left[ \mathrm{Ca}^{2+} \right]_{\mathrm{hf}}}{dt}=H_{b1}\cdot\left[ \mathrm{Ca}^{2+} \right]_{\mathrm{if}}\cdot\left( B_{H}-\left[ \mathrm{Ca}^{2+} \right]_{\mathrm{hf}} \right)-H_{b2}\cdot\left[ \mathrm{Ca}^{2+} \right]_{\mathrm{hf}} (\left[ \mathrm{Ca}^{2+} \right]_{\mathrm{hf}}\left( 0 \right)=30)$$

*V*_is_ = 1.5664$\times$10^-14^ L, *V*_if_ = 2.3496$\times$10^-14^ L, *D*_Ca_ = 6$\times$10^-8^ dm^2^ s^-1^, δ = 3$\times$10^-5^ dm, S_1_ = 3.142$\times$10^-8^ dm^2^, *L*_b1_ = 0.4 s^-1^ μM^-1^, *L*_b2_ = 0.21 s^-1^, *H*_b1_ = 100 s^-1^ μM^-1^, *H*_b2_ = 90 s^-1^, *B*_L_ = 500 μM, *B*_H_ = 300 μM

**Cyclic nucleotide-gated ion channel (*I*_CNG_ [pA])**

We adopted a comprehensive model of the phototransduction cascade complexes, which has been widely described (*see* Hamer et al. 2005, Dell'Orco et al. 2009, Invergo et al. 2014, Dell'Orco & Dal Cortivo 2019, Beelen et al. 2021). The relative ion permeability *P*_Ca_/*P*_M_ of *I*_CNG_ is about 6 (Kaupp & Seifert 2002), with *P*_M_ being the permeability of monovalent cations, including; K^+^ and Na^+^.

$$\frac{d\left[ \mathrm{cGMP} \right]}{dt}=\frac{A_{\max}}{1+\left( \frac{\left[ \mathrm{Ca}^{2+} \right]_{\mathrm{os}}}{0.171} \right)^{3}}+\frac{A_{\max}}{1+\left( \frac{\left[ \mathrm{Ca}^{2+} \right]_{\mathrm{os}}}{0.059} \right)^{1.5}}-\left[ \mathrm{cGMP} \right]\cdot\left( \beta_{\mathrm{dark}}+\beta_{\mathrm{sub}}\cdot\left[ \mathrm{PDE} \right]^{*} \right)$$

Hamer et al. (2005) showed the equation (Eq. A12 in Hamer et al. 2005) of *I*_CNG_ as the following equation.

$$I_{\mathrm{CNG}}=-\frac{2}{2+f_{\mathrm{Ca}}}\cdot J_{\mathrm{dark}}\cdot\left( \frac{\left[ \mathrm{cGMP} \right]}{\left[ \mathrm{cGMP} \right]_{\mathrm{dark}}} \right)^{n_{\mathrm{CG}}}$$

*I*_CNG_ is the total of ionic current derived from Ca^2+^, Na^+^, and K^+^ and given by

$$I_{\mathrm{CNG}}=I_{CNG,Ca}+I_{CNG,Na}+I_{CNG,K}$$

$$I_{CNG,Ca}=P_{Ca,CNG}\cdot C_{F,Caos}\cdot p_{Open,CNG}\cdot C_{m}$$

$$I_{CNG,Na}=P_{Na,CNG}\cdot C_{F,Na}\cdot p_{Open,CNG}\cdot C_{m}$$

$$I_{CNG,K}=P_{K,CNG}\cdot C_{F,K}\cdot p_{Open,CNG}\cdot C_{m}$$

$$I_{\mathrm{CNG}}=p_{Open,CNG}\cdot C_{m}\cdot(P_{Ca,CNG}\cdot C_{F,Caos}+P_{Na,CNG}\cdot C_{F,Na}+P_{K,CNG}\cdot C_{F,K})$$

Eq. A12 (for *I*_CNG_) in Hamer et al. (2005) equals the above *I*_CNG_ equation based on the GHK constant field equation.

$$-\frac{2}{2+f_{\mathrm{Ca}}}\cdot J_{\mathrm{dark}}\cdot\left( \frac{\left[ \mathrm{cGMP} \right]}{\left[ \mathrm{cGMP} \right]_{\mathrm{dark}}} \right)^{n_{\mathrm{CG}}}=p_{Open,CNG}\cdot C_{m}\cdot(P_{Ca,CNG}\cdot C_{F,Caos}+P_{Na,CNG}\cdot C_{F,Na}+P_{K,CNG}\cdot C_{F,K})$$

Finally, we obtained the open probability of *I*_CNG_ in the new model.

$$p_{Open,CNG}=-\frac{2}{2+f_{\mathrm{Ca}}}\cdot J_{\mathrm{dark}}\cdot\left( \frac{\left[ \mathrm{cGMP} \right]}{\left[ \mathrm{cGMP} \right]_{\mathrm{dark}}} \right)^{n_{\mathrm{CG}}}\cdot\frac{1}{C_{m}\cdot(P_{Ca,CNG}\cdot C_{F,Caos}+P_{Na,CNG}\cdot C_{F,Na}+P_{K,CNG}\cdot C_{F,K})}$$

*A*_max_ = 60 μM s^-1^, $\beta_{\mathrm{dark}}$= 3.1873 s^-1^, $\beta_{\mathrm{sub}}$ = 0.0022 s^-1^ μM^-1^, *f*_Ca_ = 0.12, [cGMP]_dark_ = 6.4944 μM, *J*_dark_ = 14.87 pA

**Potassium dependent sodium/calcium exchanger (*I*_NCKX_ [pA])**

*I*_NCKX_ from Hamer et al. (2005)

$$I_{\mathrm{NCKX}}=-\frac{f_{\mathrm{Ca}}}{2+f_{\mathrm{Ca}}}\cdot J_{\mathrm{dark}}\cdot\frac{\left[ \mathrm{Ca}^{2+} \right]_{\mathrm{os}}-\left[ \mathrm{Ca}^{2+} \right]_{0}}{\left[ \mathrm{Ca}^{2+} \right]_{os,dark}-\left[ \mathrm{Ca}^{2+} \right]_{0}}$$

$\left[ \mathrm{Ca}^{2+} \right]_{os,dark}$ = 0.25 μM, $\left[ \mathrm{Ca}^{2+} \right]_{0}$ = 0.023 μM

**Model fitting for ion permeability of *I*_CNG_ based GHK equation in the current model**

In the darkness, Ca^2+^ influx is equal to Ca^2+^ efflux at rod outer segments.

$$-P_{Ca,CNG}\cdot C_{F,Caos}\cdot p_{Open,CNG}\cdot C_{m}\cdot\frac{1}{2\cdot F\cdot V_{\mathrm{os}}}=-\frac{f_{\mathrm{Ca}}}{2+f_{\mathrm{Ca}}}\cdot J_{\mathrm{dark}}\cdot\frac{\left[ \mathrm{Ca}^{2+} \right]_{\mathrm{os}}-\left[ \mathrm{Ca}^{2+} \right]_{0}}{\left[ \mathrm{Ca}^{2+} \right]_{os,dark}-\left[ C\mathrm{Ca}^{2+}a \right]_{0}}\cdot\frac{-2}{2\cdot F\cdot V_{\mathrm{os}}}$$

$$-P_{Ca,CNG}\cdot C_{F,Caos}\cdot p_{Open,CNG}\cdot C_{m}=-\frac{f_{\mathrm{Ca}}}{2+f_{\mathrm{Ca}}}\cdot J_{\mathrm{dark}}\cdot\frac{\left[ \mathrm{Ca}^{2+} \right]_{\mathrm{os}}-\left[ \mathrm{Ca}^{2+} \right]_{0}}{\left[ \mathrm{Ca}^{2+} \right]_{os,dark}-\left[ \mathrm{Ca}^{2+} \right]_{0}}\cdot(-2)$$

Replace *p*_Open,CNG_;

$$-P_{Ca,CNG}\cdot C_{F,Caos}\cdot\left( -\frac{2}{2+f_{\mathrm{Ca}}}\cdot J_{\mathrm{dark}}\cdot\left( \frac{\left[ \mathrm{cGMP} \right]}{\left[ \mathrm{cGMP} \right]_{\mathrm{dark}}} \right)^{n_{\mathrm{CG}}}\cdot\frac{1}{C_{m}\cdot\left( P_{Ca,CNG}\cdot C_{F,Caos}+P_{Na,CNG}\cdot C_{F,Na}+P_{K,CNG}\cdot C_{F,K} \right)} \right)\cdot C_{m} =-\frac{f_{\mathrm{Ca}}}{2+f_{\mathrm{Ca}}}\cdot J_{\mathrm{dark}}\cdot\frac{\left[ \mathrm{Ca}^{2+} \right]_{\mathrm{os}}-\left[ \mathrm{Ca}^{2+} \right]_{0}}{\left[ \mathrm{Ca}^{2+} \right]_{os,dark}-\left[ \mathrm{Ca}^{2+} \right]_{0}}\cdot(-2)$$

$$P_{Ca,CNG}\cdot C_{F,Caos}\cdot\left( \frac{\left[ \mathrm{cGMP} \right]}{\left[ \mathrm{cGMP} \right]_{\mathrm{dark}}} \right)^{n_{\mathrm{CG}}}\cdot\frac{1}{P_{Ca,CNG}\cdot C_{F,Caos}+P_{Na,CNG}\cdot C_{F,Na}+P_{K,CNG}\cdot C_{F,K}}=f_{\mathrm{Ca}}\cdot\frac{\left[ \mathrm{Ca}^{2+} \right]_{\mathrm{os}}-\left[ \mathrm{Ca}^{2+} \right]_{0}}{\left[ \mathrm{Ca}^{2+} \right]_{os,dark}-\left[ \mathrm{Ca}^{2+} \right]_{0}}$$

In the darkness; [cGMP] = [cGMP]_dark_, $\left[ \mathrm{Ca}^{2+} \right]_{\mathrm{os}}$ = $\left[ \mathrm{Ca}^{2+} \right]_{os,dark}$

$$\frac{P_{Ca,CNG}\cdot C_{F,Caos}}{P_{Ca,CNG}\cdot C_{F,Caos}+P_{Na,CNG}\cdot C_{F,Na}+P_{K,CNG}\cdot C_{F,K}}=f_{\mathrm{Ca}}$$

In the darkness; $\left[ \mathrm{Ca}^{2+} \right]_{\mathrm{os}}$= 0.25 μM, $\left[ \mathrm{Ca}^{2+} \right]_{o}$= 1600 μM, $\left[ \mathrm{Na}^{+} \right]_{i}$ = 5 mM, $\left[ \mathrm{Na}^{+} \right]_{o}$= 145 mM, $\left[ K^{+} \right]_{i}$= 140 mM, $\left[ K^{+} \right]_{o}$= 5 mM, $C_{F,Caos}$= -4.64, $C_{F,Na}$= -262.37, $C_{F,K}$ = 56.83,

$P_{\mathrm{Na}}$/$P_{K}$ = ~1, $P_{\mathrm{Ca}}$/$P_{\mathrm{Na}}$= 1.9-6.5 (Picones & Korenbrot 1995, Wells & Tanaka 1997, Hackos & Korenbrot 1999, Kaupp & Seifert 2002)

$P_{\mathrm{Na}}:P_{K}:P_{\mathrm{Ca}}$ = 1*x* : 1*x* : *R*_Ca_ *x* ; *x* = scaling factor

*R*_Ca_: 1.9 to 6.5

Fitting with *f*_Ca_ = 0.12

$$\frac{(R_{Ca}x)\cdot(-4.64)}{(R_{Ca}x)\cdot(-4.64)+(1x) \cdot(-262.37)+(1x)\cdot56.83}=0.12$$

$$\frac{-4.64\cdot R_{Ca}}{-4.64\cdot R_{Ca}-262.37+56.83}=0.12$$

$R_{Ca}=6.026$, $P_{\mathrm{Ca}}\cong6x$, *x* = 1/3.6

**L-type voltage-gated calcium channel (*I*_CaL_ [pA])**

Electrophysiological profiles; Morgans et al. 2005, Babai and Thoreson 2009, Grove et al. 2019, Ingram et al. 2020

Ion permeability; Takeuchi et al. 2006


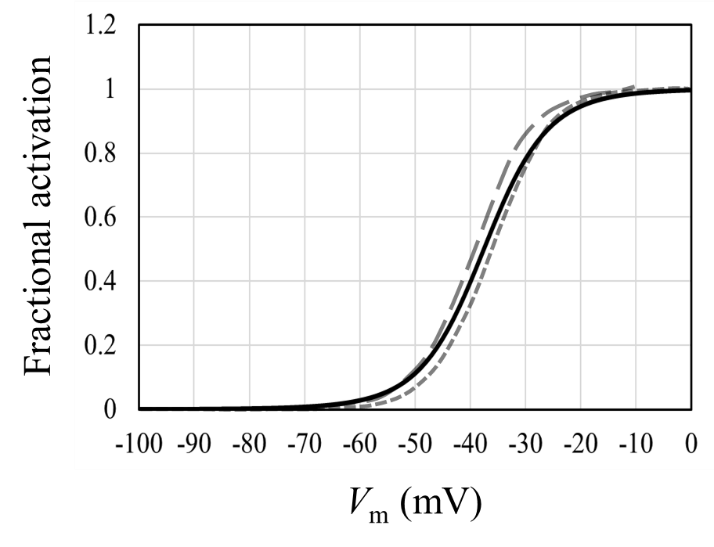


**Fig. 2S** Fractional activation of *I*_CaL_ in the current study (black line) and experimental observations. Dotted line; mouse rods (Morgans et al. 2005), Dashed line; mouse rods and cones (Babai and Thoreson 2009, Grove et al. 2019, Ingram et al. 2020)).

$$I_{\mathrm{CaL}}=I_{CaL,Ca}+I_{CaL,K}+I_{CaL,Na}$$

$$I_{\mathrm{CaL},\mathrm{Ca}}=P_{Ca,CaL}\cdot C_{F,Cais}\cdot p_{Open,CaL}\cdot C_{m}$$

$$I_{CaL,K}=P_{K,CaL}\cdot C_{F,K}\cdot p_{Open,CaL}\cdot C_{m}$$

$$I_{CaL,Na}=P_{Na,CaL}\cdot C_{F,Na}\cdot p_{Open,CaL}\cdot C_{m}$$

$$\alpha_{\mathrm{CaL}}=\frac{1000}{1+\exp\left( -\frac{V_{m}+20}{10} \right)}$$

$$\beta_{\mathrm{CaL}}=\frac{1000}{1+\exp\left( \frac{V_{m}+55}{10} \right)}$$

$$\frac{dp_{Open,CaL}}{dt}=\alpha_{\mathrm{CaL}}\cdot\left( 1-p_{Open,CaL} \right)-\beta_{\mathrm{CaL}}\cdot p_{Open,CaL} (p_{Open,CaL}\left( 0 \right)=0.556)$$

*k*_CaL_ = 0.65 (scale factor), *P*_Ca,CaL_ = 1$\times$ *k*_CaL_, *P*_K,CaL_ = 0.000365$\times$ *k*_CaL_, *P*_Na,CaL_ = 0.0000185$\times$ *k*_CaL_

**Plasma membrane Ca^2+^ pump (*I*_PMCA_ [pA])**

Model equations; Takeuchi et al. 2006 & Kamiyama et al. 2009


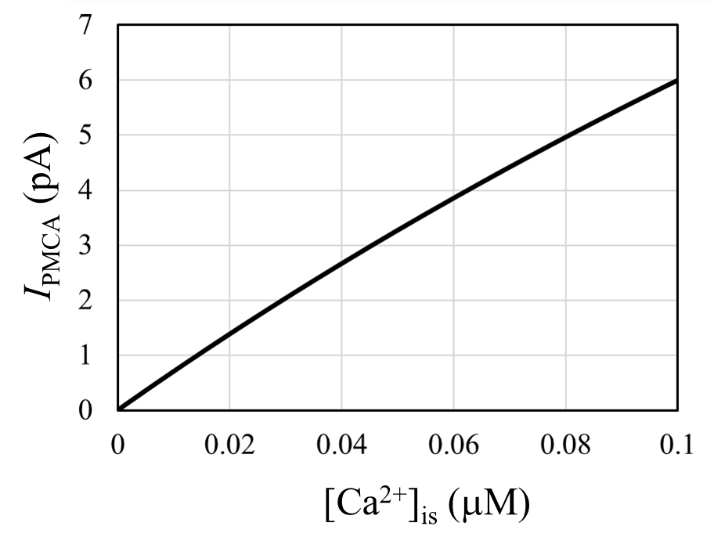


**Fig. 3S** Electrophysiological characteristics of *I*_PMCA_ in the current study.

$$I_{\mathrm{PMCA}}=\frac{k_{\mathrm{PMCA}}}{1+\frac{0.0005}{\left[ \mathrm{Ca} \right]_{\mathrm{is}}\cdot{10}^{-3}}}\cdot C_{m}$$

*k*_PMCA_ = 10

**Hyperpolarization-activated cyclic nucleotide-gated ion channel (*I*_h_ [pA])**

Model equations; Kamiyama et al. 2009

Model fitting for shaping membrane potential response to light, i.e., characteristics of peak-plateau sag.


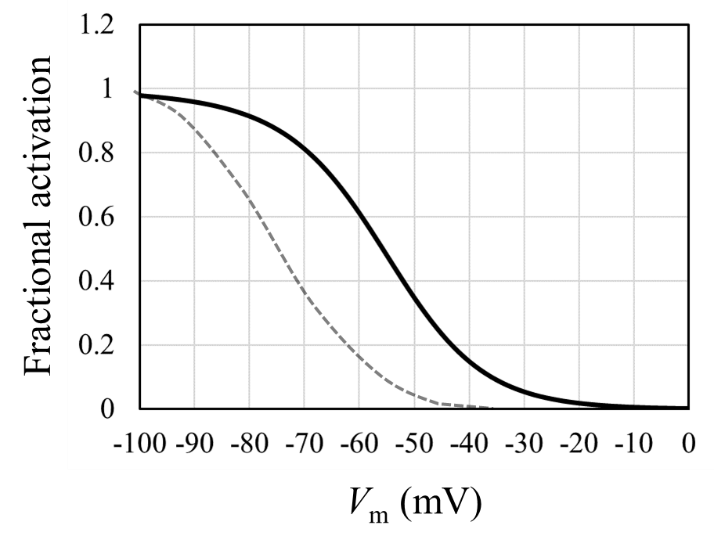


**Fig. 4S** Fractional activation of *I*_h_ in the current study (black line) and rabbit rod (Dotted line) from Demontis et al. (2002).

$$I_{h}=I_{h,K}+I_{h,Na}$$

$$I_{h,K}=P_{K,h}\cdot C_{F,K}\cdot p_{Open,h}\cdot C_{m}$$

$$I_{h,Na}=P_{Na,h}\cdot C_{F,Na}\cdot p_{Open,h}\cdot C_{m}$$

$$\alpha_{h}=\frac{80}{1+\exp\left( \frac{V_{m}+78}{14} \right)}$$

$$\beta_{h}=\frac{180}{1+\exp\left( -\frac{V_{m}+8}{19} \right)}$$

$$\frac{dp_{Open,h}}{dt}=\alpha_{h}\cdot\left( 1-p_{Open,h} \right)-\beta_{h}\cdot p_{Open,h} (p_{Open,h}\left( 0 \right)=0.103)$$

*P*_K,h_ = 0.15, *P*_Na,h_ = 0.05

**Voltage-gated potassium channel** **(*I*_Kv_ [pA])**

Model equations; Fortenbach et al. 2021


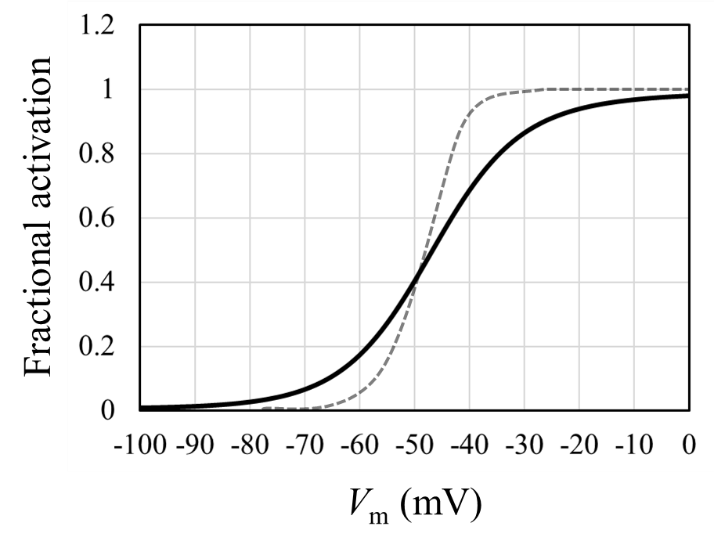


**Fig. 5S** Fractional activation of *I*_Kv_ in the current study (black line) and monkey rod (Dotted line) from Gayet-Primo et al. (2018).

$$I_{\mathrm{Kv}}=g_{\mathrm{Kv}}\cdot p_{Open,Kv}\cdot(V_{m}-E_{K})$$

$$\alpha_{\mathrm{Kv}}=0.5+\frac{30-0.5}{1+\exp\left( -\frac{V_{m}-5}{17} \right)}$$

$$\beta_{\mathrm{Kv}}=0.25+\frac{100-0.25}{1+\exp\left( \frac{V_{m}+92}{11} \right)}$$

$$\frac{dp_{Open,Kv}}{dt}=\alpha_{\mathrm{Kv}}\cdot\left( 1-p_{Open,Kv} \right)-\beta_{\mathrm{Kv}}\cdot p_{Open,Kv} (p_{Open,Kv}\left( 0 \right)=0.769)$$

*g*_Kv_ = 0.2 nS

**Calcium-activated potassium channel (*I*_KCa_ [pA])**

Model equations; Kamiyama et al. 2009


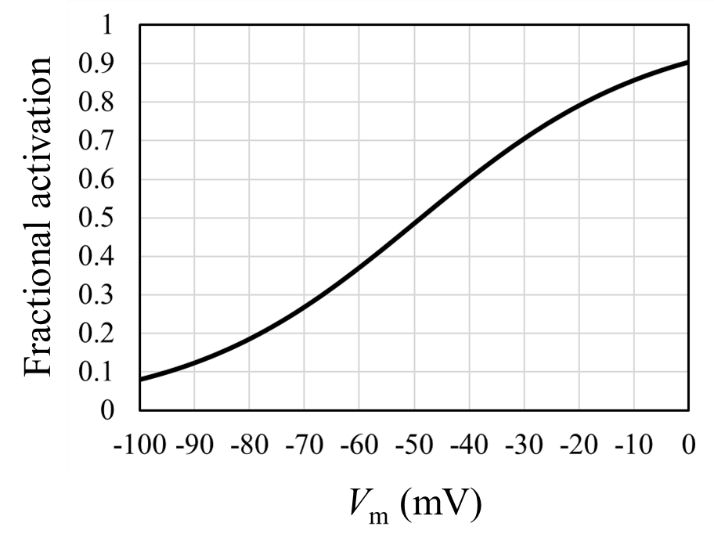


**Fig. 6S** Fractional activation of *I*_KCa_ in the current study.

$$I_{\mathrm{KCa}}=g_{\mathrm{KCa}}\cdot m_{\mathrm{KCa}}^{2}\cdot m_{\mathrm{KCas}}\cdot(V_{m}-E_{K})$$

$$\alpha_{\mathrm{mKCa}}=\frac{15\cdot(80-V_{m})}{\exp\left( \frac{{80-V}_{m}}{40} \right)-1}$$

$$\beta_{\mathrm{mKCa}}=20\cdot\exp\left( -\frac{V_{m}}{35} \right)$$

$$\frac{dm_{\mathrm{KCa}}}{dt}=\alpha_{\mathrm{mKCa}}\cdot\left( 1-m_{\mathrm{KCa}} \right)-\beta_{\mathrm{mKCa}}\cdot m_{\mathrm{KCa}} (m_{\mathrm{KCa}}\left( 0 \right)=0.642)$$

$$m_{\mathrm{KCas}}=\frac{\left[ \mathrm{Ca} \right]_{\mathrm{is}}}{\left[ \mathrm{Ca} \right]_{\mathrm{is}}+0.3}$$

*g*_KCa_ = 0.113 nS

**Calcium-activated chloride channel** **(*I*_ClCa_ [pA])**

Model equations; Kamiyama et al. 2009


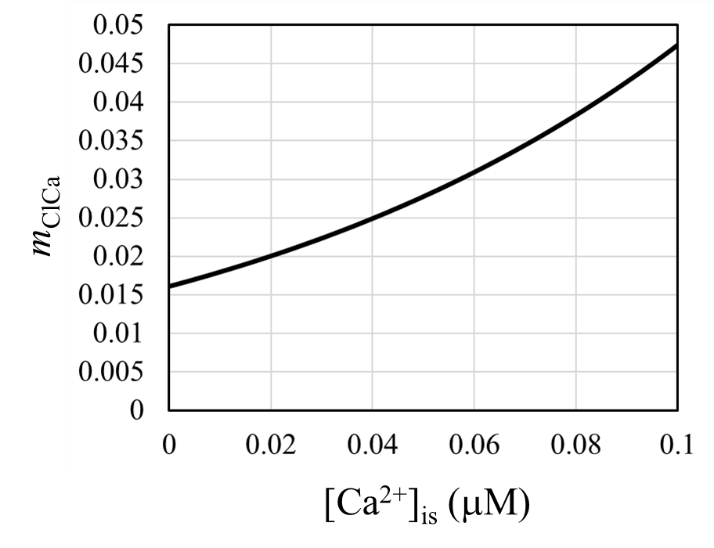


**Fig. 7S** The relationship between *m*_ClCa_ and calcium level.

$$I_{\mathrm{ClCa}}=g_{\mathrm{ClCa}}\cdot m_{\mathrm{ClCa}}\cdot(V_{m}-E_{\mathrm{Cl}})$$

$$m_{\mathrm{ClCa}}=\frac{1}{1+\exp\left( \frac{0.37-\left[ \mathrm{Ca} \right]_{\mathrm{is}}}{0.09} \right)}$$

*g*_ClCa_ = 2 nS

**Sodium/potassium pump** **(*I*_NaK_ [pA])**

Model equations; Fortenbach et al. 2021 (Original source; *see* Figure S4 in Fortenbach et al. 2021)

$$I_{\mathrm{NaK}}=10.34\cdot\left( 0.33+\frac{1-0.33}{1+\exp\left( -\frac{V_{m}+57.1}{27.6} \right)} \right)$$

**Sodium/calcium exchanger** **(*I*_NCX_ [pA])**

Model equations; Luo & Rudy 1994

$$I_{\mathrm{NCX}}=k_{\mathrm{NCX}}\cdot\frac{1}{K_{m,Na}^{3}+\left[ \mathrm{Na} \right]_{o}^{3}}\cdot\frac{1}{K_{m,Ca}+\left[ \mathrm{Ca} \right]_{o}\cdot{10}^{-3}}\cdot\frac{1}{1+k_{\mathrm{sat}}\cdot\exp\left( \left( \eta-1 \right)\cdot V_{m}\cdot\frac{F}{R\cdot T} \right)}\cdot{10}^{-3}\cdot\left( \exp\left( \eta\cdot V_{m}\cdot\frac{F}{R\cdot T} \right)\cdot\left[ \mathrm{Na} \right]_{i}^{3}\cdot\left[ \mathrm{Ca} \right]_{o}-\exp\left( \left( \eta-1 \right)\cdot V_{m}\cdot\frac{F}{R\cdot T} \right)\cdot\left[ \mathrm{Na} \right]_{o}^{3}\cdot\left[ \mathrm{Ca} \right]_{i} \right)$$

*k*_NCX_ = 3.6$\times$250 μA/μF, *K*_m,Na_ = 87.5 mM, *K*_m,Ca_ = 1.38 mM, *k*_sat_ = 0.1, $\eta$ = 0.35

**Cotransporters [mM]**

Model equations; Wei et al. 2014

$$J_{NKCC1}=-k_{NKCC1}\cdot\left( 0.1\cdot\frac{1}{1+\exp(16-\left[ K^{+} \right]_{o})}\cdot\ln\left( \frac{\left[ K^{+} \right]_{i}\cdot\left[ Cl^{-} \right]_{i}}{\left[ K^{+} \right]_{o}\cdot\left[ Cl^{-} \right]_{o}} \right)+\ln\left( \frac{\left[ Na^{+} \right]_{i}\cdot\left[ Cl^{-} \right]_{i}}{\left[ Na^{+} \right]_{o}\cdot\left[ Cl^{-} \right]_{o}} \right) \right)$$

$$J_{KCC2}=-k_{KCC2}\cdot0.3\cdot\ln\left( \frac{\left[ K^{+} \right]_{i}\cdot\left[ Cl^{-} \right]_{i}}{\left[ K^{+} \right]_{o}\cdot\left[ Cl^{-} \right]_{o}} \right)$$

*k*_NKCC1_ = 0.005, *k*_KCC2_ = 0.046

**Unidentified leak channels** **(*I*_L_ [pA])**

Leak channels of four essential ion species

$$I_{L,K}=g_{L,K}\cdot(V_{m}-E_{K})$$

$$I_{L,Na}=g_{L,Na}\cdot\left( V_{m}-E_{\mathrm{Na}} \right)$$

$$I_{L,Cl}=g_{L,Cl}\cdot\left( V_{m}-E_{\mathrm{Cl}} \right)$$

$$I_{L,Cais}=g_{L,Cais}\cdot\left( V_{m}-E_{\mathrm{Cais}} \right)$$

$$I_{L,Caos}=g_{L,Caos}\cdot(V_{m}-E_{\mathrm{Caos}})$$

*g*_L,K_ = 0.00011488937611594242

*g*_L,Na_ = 0.000071150566012260358

*g*_L,Cl_ = 0.00035986965484864007

*g*_L,Cais_ = 0.0002481452439628276

*g*_L,Caos_ = 0.000021232320024139139

**Ion concentration**

$$\frac{d\left[ K^{+} \right]}{\mathrm{dt}}=\frac{-(I_{CNG,K}-1\cdot I_{\mathrm{NCKX}}+I_{h,K}+I_{\mathrm{Kv}}+I_{CaL,K}+I_{\mathrm{KCa}}-2\cdot I_{\mathrm{NaK}}+I_{L,K})}{1\cdot F\cdot V_{\mathrm{cell}}}\cdot{10}^{-9}+J_{NKCC1}+J_{KCC2}$$

$$\frac{d\left[ \mathrm{Na}^{+} \right]}{dt}=\frac{-(I_{CNG,Na}+4\cdot I_{\mathrm{NCKX}}+I_{h,Na}+I_{CaL,Na}+3\cdot I_{\mathrm{NaK}}+3\cdot I_{\mathrm{NCX}}+I_{L,Na})}{1\cdot F\cdot V_{\mathrm{cell}}}\cdot{10}^{-9}+J_{NKCC1}$$

$$\frac{d\left[ \mathrm{Cl}^{-} \right]}{dt}=\frac{-(I_{\mathrm{ClCa}}+I_{L,Cl})}{-1\cdot F\cdot V_{\mathrm{cell}}}\cdot{10}^{-9}+2\cdot J_{NKCC1}+J_{KCC2}$$

*F* = 96485.34 C mol^-1^, *V*_cell_ = 7.832$\times$10^-14^ L

**Required ATP for ion pumping**

$$\frac{d\left[ \mathrm{Na}^{+} \right]_{\mathrm{influx}}}{dt}=\frac{-(I_{CNG,Na}+4\cdot I_{\mathrm{NCKX}}+I_{h,Na}+I_{CaL,Na}+3\cdot I_{\mathrm{NCX}}+I_{L,Na})}{1\cdot F\cdot V_{\mathrm{cell}}}\cdot{10}^{-9}+J_{NKCC1}$$

$$ATP for pumping out excess Na^{+} =\frac{d\left[ \mathrm{Na}^{+} \right]_{\mathrm{influx}}}{dt}\cdot\frac{N_{A}\cdot V_{\mathrm{cell}}}{3}\cdot{10}^{-3}$$

*Note* that one ATP molecule is hydrolyzed for transporting three Na^+^ out of the cell.

$$\frac{d\left[ \mathrm{Ca}^{+} \right]_{\mathrm{influx}}}{dt}=\frac{-(I_{CaL,Ca}+I_{L,Cais}-{2\cdot I}_{\mathrm{NCX}})}{2\cdot F\cdot V_{\mathrm{is}}}\cdot{10}^{-6}$$

$$ATP for pumping out excess Ca^{2+}=\frac{d\left[ Ca^{2+} \right]_{\mathrm{influx}}}{dt}\cdot N_{A}\cdot V_{\mathrm{is}}\cdot{10}^{-6}$$

*Note* that *I*_PMCA_ consumes one ATP for pumping one Ca^2+^ out of the cell, whereas extruding one Ca^2+^ from the cell in exchange for three Na^+^ entering the cell via *I*_NCX_ has no ATP utilization. However, if *I*_NCX_ has a low expression in mouse rods, *I*_PMCA_ is mainly responsible for Ca^2+^ release mechanisms.

*N*_A_ = 6.022$\times$10^23^ molecules/mole

**References;** *Supplementary Materials*

1. Hamer, R. D., Nicholas, S. C., Tranchina, D., Lamb, T. D. & Jarvinen, J. L. P. Toward a unified model of vertebrate rod phototransduction. *Vis. Neurosci.* **22**, 417-436 (2005).
2. Dell'Orco, D., Schmidt, H., Mariani, S. & Fanelli, F. Network-level analysis of light adaptation in rod cells under normal and altered conditions. *Mol. Biosyst.* **5**, 1232-1246 (2009).
3. Invergo, B. M., Dell'Orco, D., Montanucci, L., Koch, K. W. & Bertranpetit, J. A comprehensive model of the phototransduction cascade in mouse rod cells. *Mol. BioSyst.* **10**, 1481-1489 (2014).
4. Dell'Orco, D. & Dal Cortivo, G. Normal GCAPs partly compensate for altered cGMP signaling in retinal dystrophies associated with mutations in *GUCA1A*. *Sci. Rep.* 9, 20105 (2019).
5. Beelen, C. J., Asteriti, S., Cangiano, L., Koch, K. W. & Dell'Orco, D. A hybrid stochastic/deterministic model of single photon response and light adaptation in mouse rods. *Comput. Struct. Biotechnol. J.* **19**, 3720-3734 (2021).
6. Kamiyama, Y., Wu, S. M. & Usui, S. Simulation analysis of bandpass filtering properties of a rod photoreceptor network. *Vis. Res.* **49**, 970-978 (2009).Kamiyama et al. 2009.
7. Fortenbach, C. *et al.* Loss of the K^+^ channel K_v_2. 1 greatly reduces outward dark current and causes ionic dysregulation and degeneration in rod photoreceptors. *J. Gen. Physiol.* **153**, e202012687 (2021).
8. Takeuchi, A. *et al.* Ionic mechanisms of cardiac cell swelling induced by blocking Na^+^/K^+^ pump as revealed by experiments and simulation. *J. Gen. Physiol.* **128**, 495-507 (2006).
9. Luo, C. H. & Rudy, Y. A dynamic model of the cardiac ventricular action potential. I. Simulations of ionic currents and concentration changes. *Circ. Res.* **74**, 1071-1096 (1994).
10. Wei, Y., Ullah, G. & Schiff, S. J. Unification of neuronal spikes, seizures, and spreading depression. *J. Neurosci.* **34**, 11733-11743 (2014).
11. Ingram, N. T., Sampath, A. P. & Fain, G. L. Voltage-clamp recordings of light responses from wild-type and mutant mouse cone photoreceptors. *J. Gen. Physiol.* **151**, 1287-1299 (2019).
12. Kaupp, U. B. & Seifert, R. Cyclic nucleotide-gated ion channels. *Physiol. Rev.* **82**, 769-824 (2002).
13. Picones, A. & Korenbrot, J. I. Permeability and interaction of Ca^2+^ with cGMP-gated ion channels differ in retinal rod and cone photoreceptors. *Biophys. J.* **69**, 120-127 (1995).
14. Wells, G. B. & Tanaka, J. C. Ion selectivity predictions from a two-site permeation model for the cyclic nucleotide-gated channel of retinal rod cells. *Biophys. J.* **72**, 127-140 (1997).
15. Hackos, D. H. & Korenbrot, J. I. Divalent cation selectivity is a function of gating in native and recombinant cyclic nucleotide–gated ion channels from retinal photoreceptors. *J. Gen. Physiol.* **113**, 799-818 (1999).
16. Morgans, C. W. *et al.* Photoreceptor calcium channels: insight from night blindness. *Vis. Neurosci.* **22**, 561-568 (2005).
17. Babai, N. & Thoreson, W. B. Horizontal cell feedback regulates calcium currents and intracellular calcium levels in rod photoreceptors of salamander and mouse retina. *J. Physiol.* **587**, 2353-2364 (2009).
18. Grove, J. C. *et al.* Novel hybrid action of GABA mediates inhibitory feedback in the mammalian retina. *PLoS Biol.* **17**, e3000200 (2019).
19. Ingram, N. T., Sampath, A. P. & Fain, G. L. Membrane conductances of mouse cone photoreceptors. *J. Gen. Physiol.* **152**, e201912520 (2020).
20. Demontis, G. C. *et al.* Functional characterisation and subcellular localisation of HCN1 channels in rabbit retinal rod photoreceptors. *J. Physiol.* **542**, 89-97 (2002).
21. Gayet-Primo, J., Yaeger, D. B., Khanjian, R. A. & Puthussery, T. Heteromeric K_V_2/K_V_8. 2 channels mediate delayed rectifier potassium currents in primate photoreceptors. *J. Neurosci.* **38**, 3414-3427 (2018).
